# Multidisciplinary Team Support for Patients With Head and Neck Cancer Receiving Radiotherapy: A Randomized Clinical Trial

**DOI:** 10.1001/jamanetworkopen.2025.47590

**Published:** 2025-12-15

**Authors:** Yiyan Pei, Jingjing Wang, Juejin Li, Ye Chen, Jinlan He, Zhigong Wei, Zheran Liu, Yonglin Su, Tingting Dai, Li Yin, Yaotiao Deng, Jitao Zhou, Hangrui Tian, Yunhuan Li, Xiaoli Chen, Shu Zhang, Yang Chen, Qianwen Yan, Ruidan Li, Zheng Jiang, Xiaolin Hu, Xingchen Peng

**Affiliations:** 1 Department of Biotherapy, Cancer Center, West China Hospital, Sichuan University, Chengdu, Sichuan, China; 2 Sichuan Provincial Key Laboratory of Nuclear Physics and Medical Research, Sichuan University, Chengdu, Sichuan, China; 3 Department of Oncology, West China School of Public Health and West China Fourth Hospital, Sichuan University, Chengdu, Sichuan, China; 4 Department of Nursing, West China Hospital, Sichuan University/West China School of Nursing, Sichuan University, Chengdu, Sichuan, China; 5 Department of Radiation Oncology, Cancer Center, West China Hospital, Sichuan University, Chengdu, Sichuan, China; 6 Department of Head and Neck Oncology, Cancer Center, West China Hospital, Sichuan University, Chengdu, Sichuan, China; 7 Department of Rehabilitation Medicine, West China Hospital, Sichuan University, Chengdu, Sichuan, China; 8 Department of Clinical Nutrition, West China Hospital, Sichuan University, Chengdu, Sichuan, China; 9 Mental Health Center, West China Hospital, Sichuan University, Chengdu, Sichuan, China; 10 Department of Stomatology, West China School of Public Health and West China Fourth Hospital, Sichuan University, Chengdu, Sichuan, China; 11 Tianfu Jincheng Laboratory, City of Future Medicine, Chengdu, Sichuan, China

## Abstract

**Importance:**

Guidelines recommend nutritional, psychological, and rehabilitation support for patients with head and neck cancer during radiotherapy, but many patients, particularly in China, lack efficient access to such services.

**Objective:**

To assess the effectiveness of Supportive Holistic Interventions by Nurses and Experts via Multidisciplinary Team (SHINE-MDT), a framework to coordinate nutritional, psychological, and rehabilitation support, at reducing radiotherapy interruptions, improving nutritional status, alleviating psychological burdens, and enhancing the quality of life (QOL) among patients with head and neck cancer undergoing radiotherapy.

**Design, Setting, and Participants:**

A randomized clinical trial (RCT) of SHINE-MDT vs usual care (UC) was conducted at West China Hospital from April 27 to December 1, 2023. Adult patients aged 18 years or older with head and neck cancer undergoing radiotherapy were enrolled.

**Interventions:**

Eligible patients were randomly allocated (1:1) to either UC or SHINE-MDT. The SHINE-MDT group received nutritional, psychological, and rehabilitation support delivered by a team including oncology nurse specialists, radiation and medical oncologists, dietitians, psychotherapists, and rehabilitation physicians. The UC group received supportive measures aligned with standard practice.

**Main Outcomes and Measures:**

The primary outcome was the radiotherapy interruption rate in the intent-to-treat population. Secondary outcomes included patient-reported QOL assessed using the European Organisation for Research and Treatment of Cancer QOL Questionnaire–Core 30 (QLQ-C30). Nutritional status was evaluated using Nutrition Risk Screening 2002 (NRS-2002) and the Patient-Generated Subjective Global Assessment (PG-SGA) and psychological status using a distress thermometer (DT), the Patient Health Questionnaire–9 (PHQ-9), and the Hospital Anxiety and Depression Scale (HADS). Assessments from baseline through 6 months posttreatment were analyzed using mixed-effects models for intergroup comparisons.

**Results:**

A total of 233 patients were enrolled (mean [SD] age, 51.7 [13.9] years; mean [SD] body mass index [calculated as weight in kilograms divided by height in meters squared], 23.8 [3.3]; 158 males [67.8%]). The radiotherapy interruption rate in the SHINE-MDT group was 13.9 (95% CI, 4.2-23.6) percentage points lower than that in the UC group (11.1% [95% CI, 6.0%-18.2%] vs 25.0% [95% CI, 17.2%-34.2%]; *P* = .003). The SHINE-MDT group had superior outcomes compared with the UC group at the end of radiotherapy, with better global health status (mean QLQ-C30 score, 68.59 [95% CI, 65.83-71.35] vs 64.06 [95% CI, 61.48-66.64]; *P* = .009), improved mean psychological status scores (DT: 3.02 [95% CI, 2.68-3.35] vs 4.30 [95% CI, 4.00-4.59]; HADS-Anxiety: 4.96 [95% CI, 4.27-5.66] vs 7.27 [95% CI, 6.44-8.12]; HADS-Depression: 4.48 [95% CI, 4.00-4.98] vs 6.06 [95% CI, 5.42-6.70]; PHQ-9: 2.22 [95% CI, 1.73-2.72] vs 3.49 [95% CI, 2.94-4.03]; all *P* < .001), and better nutritional indicators reflected by lower mean NRS-2002 (2.19 [95% CI, 2.07-2.31] vs 2.80 [95% CI, 2.62-2.96]) and PG-SGA (6.89 [95% CI, 6.17-7.61] vs 10.19 [95% CI, 9.27-11.11]) (both *P* < .001) scores.

**Conclusions and Relevance:**

In this RCT, the SHINE-MDT intervention reduced radiotherapy interruptions, improved nutritional status, alleviated psychological burdens, and elevated QOL, indicating this framework may improve the holistic well-being of patients with head and neck cancer receiving radiotherapy.

**Trial Registration:**

ClinicalTrials.gov Identifier: NCT05828004

## Key Points

**Question:**

Does the Supportive Holistic Interventions by Nurses and Experts via Multidisciplinary Team (SHINE-MDT) intervention to manage nutritional and psychological symptoms reduce radiotherapy interruptions for patients with head and neck cancer compared with usual care?

**Findings:**

In this randomized clinical trial of 233 patients in China, those receiving SHINE-MDT had a significantly lower radiotherapy interruption rate (11%) than the usual care group (25%).

**Meaning:**

The SHINE-MDT intervention may enhance radiotherapy delivery and patient well-being by addressing critical supportive care needs, demonstrating the value of holistic management for head and neck cancer.

## Teaser

This randomized clinical trial investigates whether a multidisciplinary collaboration of medical specialists providing nutritional and psychological support to patients with head and neck cancer can reduce radiotherapy interruption rates and improve quality of life compared with usual care.

## Introduction

Radiotherapy remains a cornerstone treatment for malignant head and neck tumors.^1^ Substantial evidence indicates that unplanned radiotherapy interruption is a robust independent predictor of poorer survival outcomes across various cancers.^2,3^ This association is particularly pronounced in patients with head and neck cancer, where studies indicate that each day of interruption reduces tumor control by at least 1.4%.^4^ Of note, 23.5% to 42.5% of patients experience such delays, with malnutrition and psychological distress identified as the primary contributing factors.^3,4,5,6^

During radiotherapy for head and neck malignant tumors, 56% to 88% of patients experience weight loss (≥5% of body weight).^7,8,9^ In 1 study, the median weight loss in these patients was significantly greater than in patients with other types of malignant tumors (2.6 kg vs 0.27 kg; *P* < .05).^10^ Moreover, 71.5% of patients receiving radiotherapy for head and neck tumors experience varying degrees of anxiety, depression, and other psychological disorders.^11^ These factors collectively establish a detrimental cycle between nutrition and psychological well-being, which may interrupt radiotherapy and impact the patient’s quality of life (QOL).^12^

These challenges are particularly acute in China, where patients often receive radiotherapy in an outpatient setting, while nutrition clinics and psychological clinics are available only in some larger-scale hospitals, limiting patients’ access to essential support. Moreover, both oncologists and patients often underestimate the importance of nutritional and psychological care, resulting in delayed interventions. These circumstances can lead to the interruption of radiotherapy, thus potentially impairing patients’ tumor control efficacy and adversely impacting long-term survival outcomes.^3,13^

Therefore, we developed Supportive Holistic Interventions by Nurses and Experts via Multidisciplinary Team (SHINE-MDT), a collaborative framework comprising oncology nurse specialists, radiation oncologists, medical oncologists, dietitians, psychotherapists, and rehabilitation physicians. The framework leverages nurses’ strengths in early symptom detection and timely management.^14^ Simultaneously, it coordinates multidisciplinary resources to deliver nutritional, psychological, and rehabilitation support for patients with head and neck cancer from the initiation of radiotherapy to 6 months posttreatment. We hypothesized that this SHINE-MDT approach would reduce radiotherapy interruption rates, improve patients’ nutritional and psychological status, and elevate their QOL compared with usual care.

## Methods

### Trial Design

This randomized clinical trial was conducted at West China Hospital, Sichuan University, Sichuan, China, from April 27 to December 1, 2023. It aimed to evaluate the effect of SHINE-MDT on the rate of radiotherapy interruption and QOL in patients undergoing radiotherapy for malignant head and neck tumors. The ethics committee on biomedical research of West China Hospital, Sichuan University, approved the trial. Written informed consent was obtained from all participants. The full study protocol is detailed in Supplement 1, with a schematic workflow provided in eFigures 1 and 2 in Supplement 2. The study followed the Consolidated Standards of Reporting Trials (CONSORT) reporting guideline.

### Participants

#### Inclusion and Exclusion Criteria

Eligible patients had pathologically confirmed malignant head and neck tumors without distant metastasis, were aged 18 years or older, and had a baseline Eastern Cooperative Oncology Group performance status ranging from 0 to 2. They were scheduled to undergo either postoperative adjuvant radiotherapy or definitive radiotherapy, with or without concurrent chemotherapy. They demonstrated adequate cognitive and literacy abilities, could complete the required questionnaires, and were willing to sign the informed consent form. Exclusion criteria included presence of other malignant tumors aside from those of the head and neck, a history of prior head and neck radiotherapy, mental illness or cognitive impairments, or uncontrolled systemic diseases that could significantly affect QOL.

#### Randomization and Masking

All patients were randomly assigned in a 1:1 ratio to 1 of 2 groups: the usual oncology care (UC) group or the SHINE-MDT group. The randomization process was done using a computer-generated random assignment list based on a randomized permutation block design. Allocation concealment was maintained by a research coordinator not involved in recruitment or intervention delivery. Details regarding the methods of random assignment and group labeling are provided in the trial protocol in Supplement 1.

#### Contamination Control

To control contamination between the 2 cohorts, several measures were implemented, including spatial segregation, temporal isolation, and the separation of key personnel. Specifically, the treating physicians responsible for routine oncology care were not part of the SHINE-MDT intervention to prevent the crossover of management strategies. Furthermore, physicians, technicians, and nursing staff adhered to consistent institutional protocols, with all major radiotherapy equipment maintained according to standardized calibration criteria. The specifics of all these measures are detailed in the protocol in Supplement 1.

#### Anticancer Treatment Regimen

All enrolled patients received radiotherapy delivered in daily fractions of 1.8 to 2.2 Gy, administered 5 times per week for 6 to 6.5 weeks, resulting in a total dose of 60 to 72 Gy to the planning target volume. The clinical target volume and organs at risk were delineated in accordance with consensus guidelines.^15,16^ Concurrent chemotherapy consisted of cisplatin, 100 mg/m^2^ every 3 weeks.

#### SHINE-MDT Interventions

SHINE-MDT was designed to provide holistic evaluation and management for patients. The team comprised risk-stratified (RS) nurses, radiation oncologists, medical oncologists, dietitians, psychotherapists, and rehabilitation physicians. SHINE-MDT provided individualized, immediate, and holistic (including nutrition, psychology, and rehabilitation) interventions based on each patient’s specific needs.

Enrolled patients underwent assessments conducted by nurses. To ensure objectivity, the nurses administering the questionnaires were blinded to the group assignments. The nutritional assessment included the Nutrition Risk Screening 2002 (NRS-2002; score range, 0-7, with higher scores indicating greater nutritional risk) and Patient-Generated Subjective Global Assessment (PG-SGA; score range, 0 or higher, with higher scores indicating more severe malnutrition). The psychological assessment comprised a distress thermometer (DT; score range, 0-10, with higher scores indicating higher levels of distress), the Hospital Anxiety and Depression Scale (HADS; score range, 0-21 for each subscale, with higher scores indicating more severe anxiety or depression symptoms), and the Patient Health Questionnaire–9 (PHQ-9; score range, 0-27, with higher scores indicating greater depression severity). Quality of life (QOL) was measured using the European Organisation for Research and Treatment of Cancer (EORTC) Quality-of-Life Questionnaire–Core 30 (QLQ-C30) and Quality of Life Questionnaire–Head and Neck Cancer Module (QLQ-H&N35). Both instruments yield standardized scores on a scale of 0 to 100; for the QLQ-C30, higher scores on functioning scales reflect better functioning, whereas higher scores on symptom scales indicate more severe symptom burden, and for the QLQ-H&N35, higher scores correspond to more severe symptoms or problems. Assessments were conducted at baseline (before radiotherapy) and weekly from radiotherapy initiation through the first month posttreatment, followed by evaluations at 2, 3, and 6 months posttreatment. Specific frequencies are detailed in Supplement 1. Based on questionnaire results, RS nurses stratified patients into 4 nutritional and psychological risk categories: none, mild, moderate, and severe. The criteria are shown in eFigures 2 through 5 in Supplement 1. For patients with no or mild risk, RS nurses provided guidance. Patients with moderate or severe risk were discussed at SHINE-MDT meetings for problem-solving.

#### UC Interventions

Patients assigned to the UC group received oncology care that was aligned with standard practices, including supportive measures determined by radiation oncologists and nurses. Patients routinely completed questionnaire assessments conducted by questionnaire assessment nurses. Based on the results, RS nurses offered different measures. For patients identified as having no or mild nutritional or psychological risk, RS nurses provided nutritional and psychological guidance. Patients identified with moderate or severe risk were referred to outpatient clinics for nutritional and psychological consultations, with the decision to attend the clinics left to their own discretion.

### Data Collection and Patient Outcomes

The primary end point was the radiotherapy interruption rate assessed throughout the radiotherapy course. Radiotherapy interruption was defined as 5 or more days of unplanned treatment delay (actual radiotherapy days minus planned days), regardless of the causes for interruption.^2,3^ The radiotherapy interruption rate was calculated as the proportion of individuals in each group who experienced such a delay. Weekends and statutory holidays were counted into the planned radiotherapy schedule. Secondary end points were (1) quality of life, (2) nutritional status, (3) psychological status, (4) rehospitalization rate, and (5) tumor response. *Rehospitalization* was defined as any unplanned hospital admission within 6 months after completing radiotherapy for reasons unrelated to antitumor treatment. Tumor response assessments were performed according to Response Evaluation Criteria in Solid Tumors (RECIST), version 1.1, at 3 months after completing radiotherapy.

### Statistical Analysis

Based on literature reports and clinical experience, the anticipated radiotherapy interruption rate in the control group was approximately 25%,^3,6^ compared with an anticipated 10% in the experimental group. The sample size was calculated assuming a 1-sided α level of .025 and 80% power. Accounting for an estimated 5% overall dropout rate, the required sample size was calculated as 214 participants, with 107 allocated to each group (eMethods in Supplement 2).

Radiotherapy interruption rates, rehospitalizations, and tumor response were analyzed by intention-to-treat principles, with secondary outcomes (nutrition, psychology, and QOL) assessed using per-protocol principles. Continuous variables are presented as mean with SD or median with IQR, while categorical variables are presented as number (percentage). Continuous baseline variables were compared using independent Student *t* tests, while categorical baseline variables were analyzed via χ^2^ tests. Radiotherapy interruption rates, rehospitalization rates, and tumor response rates were analyzed by χ^2^ tests. The 95% CI for each rate was calculated using the Wald method. The 95% CIs for means were calculated using the *t*-interval method. The 95% CIs for the durations of radiotherapy interruption were estimated using the nonparametric bootstrap method. A mixed-effects model was used to calculate estimated group means, mean differences, 95% CIs, and *P* values in questionnaire scores across follow-up time points and their overall effect. The model incorporated both fixed effects (treatment group, time, and group × time interaction) and random effects (participant), accounting for the correlation structure of repeated measurements. These statistical tests of results were 2-sided, with a significance level of *P* < .05. Formal multiplicity adjustment of 95% CIs and *P* values was not performed, and findings should be interpreted cautiously. Statistical analyses were conducted using R, version 4.3.1 (R Project for Statistical Computing).

## Results

### Patient Characteristics

**Figure 1.**
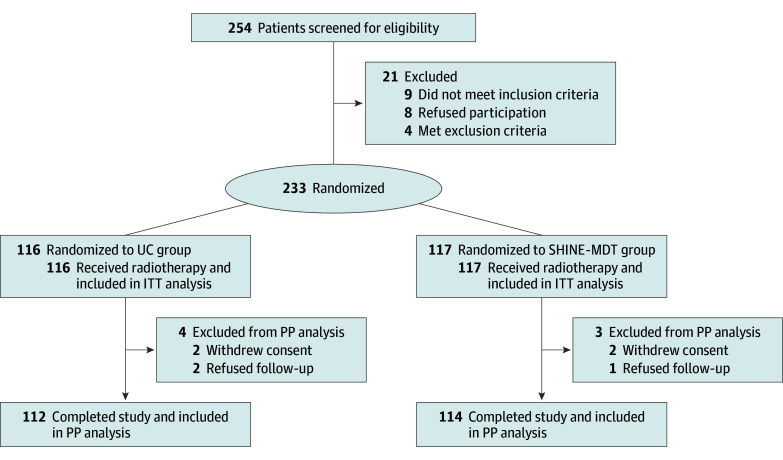
CONSORT Diagram of Participant Flow Through Trial CONSORT indicates Consolidated Standards of Reporting Trials reporting guideline; ITT, intent to treat; PP, per protocol; SHINE-MDT, Supportive Holistic Interventions by Nurses and Experts via Multidisciplinary Team; UC, usual care.

A total of 254 patients with malignant head and neck tumors were screened; 233 met the inclusion criteria and were enrolled in the trial (75 [32.2%] female, 158 [67.8%] male; mean [SD] age, 51.7 [13.9] years; mean [SD] body mass index [BMI, calculated as weight in kilograms divided by height in meters squared], 23.8 [3.3]). These patients were randomly assigned to either the UC group (n = 116) or the SHINE-MDT group (n = 117) (Figure 1). The baseline characteristics of eligible patients were balanced between the 2 groups (Table). Overall, patients in the UC group had a mean (SD) age of 52.6 (14.9) years and a mean (SD) BMI of 23.4 (3.4), while patients in the SHINE-MDT group had a mean (SD) age of 50.8 (12.9) years, with a mean BMI of 24.1 (3.2). More than half of the patients received definitive radiotherapy, including 74 (63.8%) in the UC group and 71 (60.7%) in the SHINE-MDT group. Nasopharyngeal cancer was prevalent in both groups: 65 patients (56.0%) in the UC group and 64 (54.7%) in the SHINE-MDT group. Most patients in both groups received concurrent chemotherapy: 80 (69.0%) in the UC group and 80 (68.4%) in the SHINE-MDT group. Total radiotherapy and organ-at-risk doses are summarized in eTable 1 in Supplement 2. In the UC group, 12 patients (10.3%) received psychology consultations, 17 patients (14.7%) underwent nutrition consultations, and 10 patients (8.6%) attended rehabilitation counseling throughout the entire follow-up period (eTable 2 in Supplement 2).

**Table.** Baseline Characteristics of Participants in the Study Groups

Characteristic	Participants^a^		
	UC (n = 116)	SHINE-MDT (n = 117)	Overall (n = 233)
Age, mean (SD), y	52.6 (14.9)	50.8 (12.9)	51.7 (13.9)
Sex			
Female	34 (29.3)	41 (35.0)	75 (32.2)
Male	82 (70.7)	76 (65.0)	158 (67.8)
BMI, mean (SD)	23.4 (3.4)	24.1 (3.2)	23.8 (3.3)
ECOG performance status			
0	47 (40.5)	48 (41.0)	95 (40.8)
1	62 (53.4)	61 (52.1)	123 (52.8)
2	7 (6.0)	8 (6.8)	15 (6.4)
Tobacco use			
Yes	54 (46.6)	62 (53.0)	116 (49.8)
No	62 (53.4)	55 (47.0)	117 (50.2)
Alcohol use			
Yes	46 (39.7)	60 (51.3)	106 (45.5)
No	70 (60.3)	57 (48.7)	127 (54.5)
Tumor site			
Nasopharynx	65 (56.0)	64 (54.7)	129 (55.4)
Oral cavity	22 (19.0)	22 (18.8)	44 (18.9)
Oropharynx	5 (4.3)	9 (7.7)	14 (6.0)
Hypopharynx	6 (5.2)	2 (1.7)	8 (3.4)
Larynx	3 (2.6)	7 (6.0)	10 (4.3)
Others	15 (12.9)	13 (11.1)	28 (12.0)
Tumor pathologic type			
Squamous carcinoma	102 (87.9)	98 (83.8)	200 (85.8)
Other	14 (12.1)	19 (16.2)	33 (14.2)
TNM stage^b^			
I-II	33 (28.4)	32 (27.4)	65 (27.9)
III	44 (37.9)	45 (38.5)	89 (38.2)
IVA	36 (31.0)	38 (32.4)	79 (31.8)
IVB	3 (2.6)	2 (1.7)	5 (2.1)
Tumor primary site			
T1	33 (28.4)	31 (26.5)	64 (27.5)
T2	44 (37.9)	47 (40.2)	91 (39.1)
T3	27 (23.3)	26 (22.2)	53 (22.7)
T4	12 (10.3)	13 (11.1)	25 (10.7)
Nodal involvement			
N0	29 (25.0)	22 (18.8)	51 (21.9)
N1	31 (26.7)	38 (32.5)	69 (29.6)
N2	30 (25.9)	33 (28.2)	63 (27.0)
N3	26 (22.4)	24 (20.5)	50 (21.5)
Treatment type			
Definitive	74 (63.8)	71 (60.7)	145 (62.2)
Postoperative	42 (36.2)	46 (39.3)	88 (37.8)
Concurrent chemotherapy			
Yes	80 (69.0)	80 (68.4)	160 (68.7)
No	36 (31.0)	37 (31.6)	73 (31.3)

Abbreviations: BMI, body mass index (calculated as weight in kilograms divided by height in meters squared); ECOG, Eastern Cooperative Oncology Group; SHINE-MDT, Supportive Holistic Interventions by Nurses and Experts via Multidisciplinary Team; UC, usual care.

^a^ Data are presented as number (percentage) of participants unless otherwise indicated.

^b^ Tumor staging was based on the American Joint Commission on Cancer (AJCC) Staging Manual (8th ed).

### Radiotherapy Interruption Rate

All 233 patients enrolled in the trial completed radiotherapy and were included in the analysis of radiotherapy interruptions. Among these patients, 42 (18.0%) had radiotherapy interruptions by the end of radiotherapy, including 29 (25.0%; 95% CI, 17.2%-34.2%) in the UC group and 13 (11.1%; 95% CI, 6.0%-18.2%) in the SHINE-MDT group. The radiotherapy interruption rate in the SHINE-MDT group was 13.9 (95% CI, 4.2-23.6) percentage points lower than in the UC group (*P* = .003). The duration of radiotherapy interruptions ranged from 5 to 14 days, with a median of 8.0 days (95% CI, 6.0-14.0 days) in the UC group and 8.0 days (95% CI, 5.0-12.0 days) in the SHINE-MDT group. Fatigue was the predominant reason for radiotherapy interruptions in the UC group (16 [55.2%]), whereas leukopenia accounted for most interruptions in the SHINE-MDT group (6 [46.2%]). Reasons for radiotherapy interruptions are shown in eTable 3 of Supplement 2.

### Quality of Life

**Figure 2.**
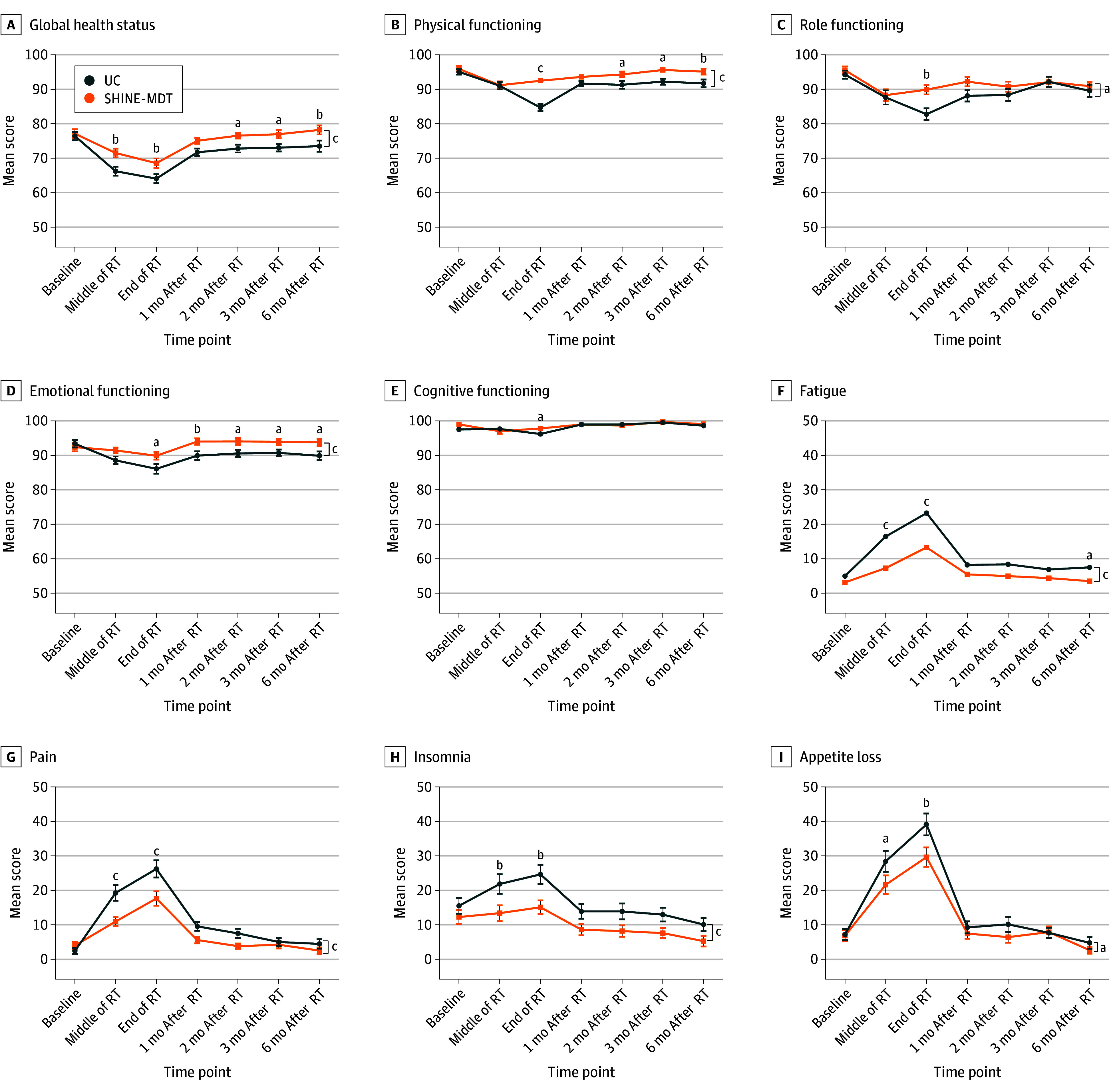
European Organisation for Research and Treatment of Cancer Quality-of-Life Questionnaire–Core 30 Assessment Scores Over Time RT indicates radiotherapy; SHINE-MDT, Supportive Holistic Interventions by Nurses and Experts via Multidisciplinary Team; UC, usual care. Whiskers represent SEs. ^a^*P* < .05. ^b^*P* < .01. ^c^*P* < .001.

**Figure 3.**
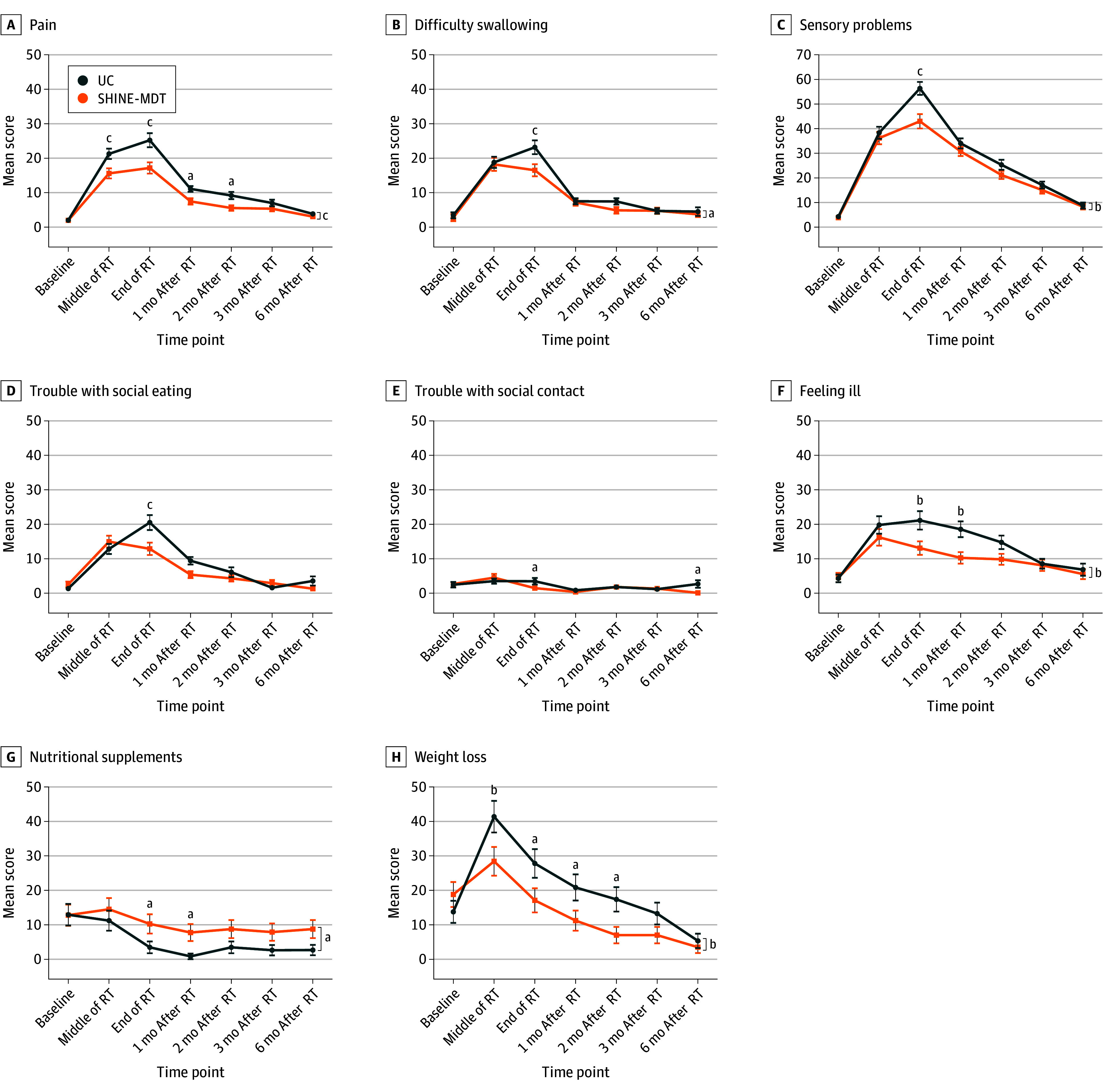
European Organisation for Research and Treatment of Cancer Quality of Life Questionnaire–Head and Neck Cancer Module Assessment Scores for Head and Neck Problems Over Time RT indicates radiotherapy; SHINE-MDT, Supportive Holistic Interventions by Nurses and Experts via Multidisciplinary Team; UC, usual care. Whiskers represent SEs. ^a^*P* < .05. ^b^*P* < .01. ^c^*P* < .001.

At the final follow-up, QOL was reported by 112 patients (96.6%) in the UC group and 114 (97.4%) in the SHINE-MDT group. Baseline scores for the EORTC QLQ-C30 and QLQ-H&N35 domains were comparable between the groups. EORTC QLQ-C30 assessments conducted at the end of radiotherapy showed superior global health status outcomes in the SHINE-MDT group compared with the UC group (mean score, 68.59 [95% CI, 65.83-71.35] vs 64.06 [95% CI, 61.48-66.64]; *P* = .009) (Figure 2A), with concurrent improvements in physical, role, emotional, and cognitive functioning outcomes (Figure 2B-E). Symptom control also favored the SHINE-MDT group, showing marked reductions in fatigue, pain, insomnia, and appetite loss (Figure 2F-I). Additionally, SHINE-MDT patients reported less severe head-and-neck–specific symptoms (eg, pain, difficulty swallowing, sensory problems, social eating problems, social contact problems, feeling ill, nutritional supplements, and weight loss) at the end of radiotherapy (Figure 3A-H). No significant differences were observed in social functioning, financial difficulties, nausea and vomiting, or other domains. Detailed scores for EORTC QLQ domains are provided in eTables 4 and 5 and eFigure 3 in Supplement 2.

### Nutritional and Psychological Status

**Figure 4.**
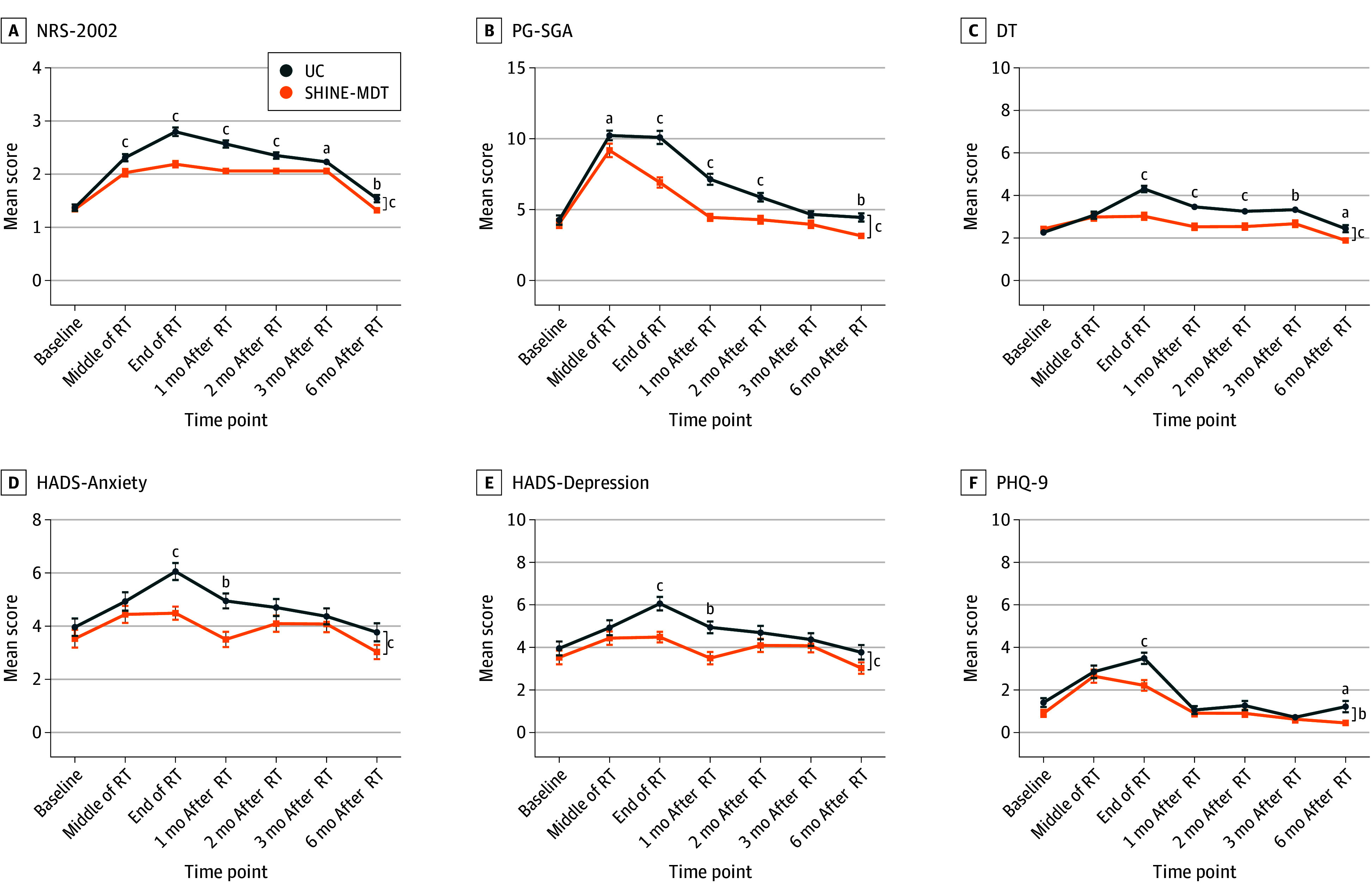
Nutritional and Psychological Assessment Scores Over Time DT indicates distress thermometer; HADS, Hospital Anxiety and Depression Scale; NRS-2002, Nutrition Risk Screening 2002; PG-SGA, Patient-Generated Subjective Global Assessment; PHQ-9, Patient Health Questionnaire–9; RT, radiotherapy; SHINE-MDT, Supportive Holistic Interventions by Nurses and Experts via Multidisciplinary Team; UC, usual care. Whiskers represent SEs. DT score range, 0 to 10, with higher scores indicating higher levels of distress; HADS score range, 0 to 21, with higher scores indicating more severe anxiety or depression symptoms; NRS-2002 score range, 0 to 7, with higher scores indicating greater nutritional risk; PG-SGA score range, 0 or higher, with higher scores indicating more severe malnutrition; PHQ-9 score range, 0 to 27, with higher scores indicating greater depression severity. ^a^*P* < .05. ^b^*P* < .01. ^c^*P* < .001.

Baseline nutritional status (NRS-2002 and PG-SGA) and psychological status (DT, HADS, and PHQ-9) were comparable between groups. Compared with the UC group, the SHINE-MDT group had superior outcomes at the end of radiotherapy, including significantly lower mean scores for nutritional evaluations (NRS-2002: 2.19 [95% CI, 2.07-2.31] vs 2.80 [95% CI, 2.62-2.96]; PG-SGA: 6.89 [95% CI, 6.17-7.61] vs 10.19 [95% CI, 9.27-11.11]; both *P* < .001) (Figure 4A-B) and psychological assessments (DT: 3.02 [95% CI, 2.68-3.35] vs 4.30 [95% CI, 4.00-4.59]; HADS-Anxiety: 4.96 [95% CI, 4.27-5.66] vs 7.27 [95% CI, 6.44-8.12]; HADS-Depression: 4.48 [95% CI, 4.00-4.98] vs 6.06 [95% CI, 5.42-6.70]; PHQ-9: 2.22 [95% CI, 1.73-2.72] vs 3.49 [95% CI, 2.94-4.03]) (all *P* < .001) (Figure 4C-F).

Longitudinal analyses showed sustained improvements in the SHINE-MDT group. Specifically, nutritional benefits included persistent reductions in NRS-2002 scores for SHINE-MDT compared with UC at all postbaseline assessments (Figure 4A) and lower PG-SGA scores at most time points (Figure 4B). The feeding-tube placement rate was notably low in both cohorts (5 patients [4.3%] in the SHINE-MDT group and 3 [2.6%] in the UC group). Weight data are provided in eTable 6 in Supplement 2. Psychological benefits of SHINE-MDT compared with UC included persistently lower DT and HADS-Anxiety scores from radiotherapy completion through 6 months (Figure 4C-D), lower HADS-Depression scores at the end of radiotherapy and at 1 month postradiotherapy (Figure 4E), and lower PHQ-9 scores at the end of radiotherapy and at 6 months postradiotherapy (Figure 4F). Detailed scores and Cronbach α values are provided in eTables 4 and 5 in Supplement 2.

### Rehospitalization Rate and Tumor Response

Upon completion of the follow-up for the last patient, 21 individuals (18.1%) from the UC group were readmitted for further treatment, compared with 9 individuals (7.7%) from the SHINE-MDT group (*P* = .02). Reasons for rehospitalization events are shown in eTable 7 in Supplement 2.

Among the enrolled patients, 88 (37.8%) received surgical treatment and 145 (62.2%) underwent definitive radiotherapy and were evaluated for tumor response. According to RECIST, version 1.1, the complete response rates were 64 of 74 (86.5%) in the UC group and 65 of 71 (91.5%) in the SHINE-MDT group, while the partial response rates were 10 of 74 (13.5%) and 6 of 71 (8.5%), respectively. No patients in either group developed stable disease or progressive disease during the follow-up period (eTable 8 in Supplement 2).

## Discussion

Patients with head and neck malignant tumors undergoing radiotherapy frequently face significant nutritional and psychological challenges, often leading to radiotherapy interruptions.^17^ However, the efficacy of an MDT approach in addressing these issues remains unclear. Building on existing evidence^7,8,9,10,11^ from nutritional and psychological interventions and clinical observations revealing periods during radiotherapy when patients are especially susceptible to nutritional and psychological burdens, we developed an MDT-based model that provides a practical and replicable framework for health care practitioners. This prospective randomized clinical trial investigated the impact of supportive interventions delivered through an MDT, revealing that SHINE-MDT effectively reduced the radiotherapy interruption rate, improved nutritional and psychological outcomes, and enhanced the QOL among patients with head and neck cancer.

Radiotherapy interruptions have been identified as an independent adverse prognostic factor in head and neck cancers, with treatment delay duration strongly correlating with diminished survival outcomes.^13,18^ Specifically, interruptions of 5 or more days have been associated with significantly reduced overall survival rates in nasopharyngeal cancer (83.4% vs 67.8%; *P* = .007).^19^ Given the probable impact of radiotherapy interruptions on patient prognosis, our study designated the radiotherapy interruption rate as the primary end point. The results demonstrated a 13.9-percentage-point absolute reduction in radiotherapy interruptions in the intervention group compared with the control group. This could be due to improved radiotherapy tolerance and treatment adherence facilitated by the SHINE-MDT intervention.

Malnutrition and psychological distress constitute 2 predominant modifiable factors associated with radiotherapy interruptions in patients with cancer.^7,17^ A previous study showed that patients at risk of malnutrition demonstrated a 5.6-fold higher radiotherapy interruption rate compared with well-nourished patients (25.2% vs 4.5%; *P* < .001).^20^ Similarly, pretreatment depression has been associated with radiotherapy interruptions and reduced survival outcomes.^21^ Given this evidence, implementing standardized nutritional and psychological screening and timely interventions into clinical workflows is essential.

The low rate of feeding tube placement (less than 10%) observed in both cohorts of this study may be attributed to 2 primary factors. First, nasopharyngeal carcinoma was highly prevalent in the study population, and this type of cancer typically requires less enteral support compared with oropharyngeal cancer.^22^ Second, cultural preferences in China tend to favor oral intake, as many patients perceive feeding tubes as invasive.^23^ This further underscores the importance of culturally sensitive counseling within the MDT framework.

Although malnutrition is a well-established prognostic factor associated with unfavorable clinical outcomes among patients with cancer, the evidence supporting the efficacy of nutritional interventions remains limited.^24^ Randomized clinical trials involving nutritional interventions demonstrated no statistically significant improvement in tumor response rates, recurrence rates, and 1- and 2-year survival rates.^25,26^ The underlying cause of these issues may be that despite implementing nutritional interventions, some patients still experience energy deficits and poor adherence, particularly patients with severe malnutrition refractory to nutritional support.^24^ In contrast, prior evidence suggests that individualized nutritional support reduces mortality risk in patients with cancer.^27^ The SHINE-MDT model addressed these limitations through tailored nutritional interventions, improving adherence through enhancing education and awareness and integrating systematic malnutrition screening for early intervention. This approach achieved sustained improvements in nutritional status.

Furthermore, considering the psychological challenges of cancer treatment, the overall prevalence of depression and anxiety among Chinese adults with malignant tumors is 54.6% and 49.69%, respectively, yet fewer than 10% receive psychological support.^28^ In our trial, a similarly low proportion (10.3%) of control group participants attended psychological counseling. In contrast, the intervention group received systematic symptom management and targeted psychological support based on individual needs. Research indicates that psychological interventions and symptom management guided by patient-reported outcomes can improve QOL, symptom control, and mood stabilization.^29,30^ These approaches may also help prevent more severe adverse events in patients with cancer.

The primary objective of an MDT is to deliver high-quality, evidence-based cancer care.^31^ Typically, an MDT comprises therapeutic specialists and diagnostic experts to provide treatment planning.^31,32,33^ However, patients undergoing radiotherapy still have unmet needs in clinical domains such as nutritional support, psychological distress management, and functional rehabilitation. In our trial, the SHINE-MDT intervention integrated nurses and experts to provide patients with holistic, individual support tailored to patient needs. Although the intervention group did not achieve statistically significant improvements in tumor response compared with the control group, a clinically meaningful trend was observed. Additionally, previous studies have shown that comprehensive supportive care, including nutritional and psychological support, may contribute to improved survival.^34^

### Strengths and Limitations

Our study has several strengths. First, the study introduced an innovation through the implementation of a protocolized questionnaire designed to guide coordinated interventions. The MDT, comprising specialists in nursing, nutrition, psychology, rehabilitation, and oncology, systematically addressed patients’ needs via this framework-structured collaboration. Second, nurses served as pivotal coordinators within this model. Beyond performing symptom monitoring and evidence-based patient education, they bridged critical communication channels between clinicians and patients while promoting interdepartmental collaboration. This approach enabled early identification and systematic resolution of emerging issues. Third, a rigorous questionnaire assessment protocol was implemented, with dedicated questionnaire assessment nurses collecting patient-reported outcomes while maintaining allocation concealment throughout the trial.

The trial also has several limitations. First, its single-center design and recruitment of an exclusively Chinese patient population may restrict the generalizability of the findings. Second, the relatively short follow-up period hindered our ability to investigate the association between SHINE-MDT and survival benefits. Future trials need to consider crucial variables that could affect results, such as radiotherapy timing. Emerging evidence points to a link between morning radiotherapy and better survival.^35^ In terms of tumor response, although no statistically significant difference was observed between groups, a trend suggesting potential clinical value was noted. Consequently, a cost-benefit analysis is warranted as a next step to assess the net benefits for optimized resource allocation.

We also acknowledge limitations in the trial’s implementation. The design necessitated the exclusion of treating physicians from the MDT; however, we maintain that in clinical practice, they should be core members. Their active engagement is critical to delivering comprehensive patient support and improving outcomes. In addition, implementing such an MDT model can be challenging in resource-limited settings with limited specialist availability. Future efforts will focus on improving accessibility via standardized practitioner training and a web-based MDT platform facilitating remote collaborative decision-making.

## Conclusions

This randomized clinical trial found that the SHINE-MDT intervention integrating nutritional support, psychological counseling, and rehabilitation services significantly reduced radiotherapy interruptions among patients with head and neck cancer. Additionally, the model enhanced nutritional status, improved psychological well-being, and elevated QOL. This research delivers a replicable framework enabling health care practitioners to improve their patients’ holistic well-being.

## Notes

Supplement 1.
Trial Protocol

Supplement 2.
eMethods
**eFigure 1.** Overview of the Study Workflow **eFigure 2.** Schematic Representation of the SHINE-MDT Intervention Flow **eFigure 3.** Quality of Life (EORTC QLQ-C30/QLQ-H&N35) Assessment Scores in UC and SHINE-MDT Groups Over Time **eTable 1.** Comparison of Radiotherapy Dose Parameters Between Treatment Groups **eTable 2.** Comparison of Consultation Recommendations and Completion Between UC Group and SHINE-MDT Group **eTable 3.** Reasons for Radiotherapy Interruptions—UC Group vs SHINE-MDT Group **eTable 4.** Longitudinal Changes in Quality of Life, Nutrition Status, and Psychological Status During Radiotherapy in Patients With Malignant Head and Neck Tumor **eTable 5.** Cronbach α Values Across Assessment Time Points **eTable 6.** Comparison of Weight Between UC and SHINE-MDT Groups During and After Radiotherapy **eTable 7.** Reasons for Rehospitalization Events—UC Group vs SHINE-MDT Group **eTable 8.** Tumor Response Between UC Group and SHINE-MDT Group

Supplement 3.
Data Sharing Statement
