# Supplementary material for: Multidisciplinary Team Support for Patients With Head and Neck Cancer Receiving Radiotherapy: A Randomized Clinical Trial
Source: JAMA Netw Open. 2025 Dec 15;8(12):e2547590. doi: 10.1001/jamanetworkopen.2025.47590 (PMC12706684; doi:10.1001/jamanetworkopen.2025.47590)
Supplement: Supplement 1. — Trial Protocol [file jamanetwopen-e2547590-s001.pdf]

Protocol

Multidisciplinary Team Support for Patients With Head and Neck Cancer Receiving Radiotherapy: A Randomized Clinical Trial

Principal Investigator: Dr. Xingchen Peng, Dr. Xiaolin Hu

Sponsor: West China Hospital, Sichuan University

1. Background ... 3

2. Objectives of the research ... 4

3. Study design, methods and procedures ... 4

4. Study assessment ... 15

5. Adverse Events and Serious Adverse Events ... 15

Appendix 1：National Cancer Institute Common Terminology Criteria for Adverse Events (NCI CTCAE) version 5.0 ... 22

Appendix 2: Eastern Cooperative Oncology Group (ECOG) - Performance Status Conversion 23

Appendix 3: Response Evaluation Criteria In Solid Tumours - RECIST 1.1 ... 24

Appendix 4: New York Heart Association (NYHA) Functional Classification ... 28

Appendix 5: Case Report Form (English version) ... 29

Appendix 6 Informed consent (English version) ... 41

1. Background

Radiotherapy (RT) serves as a principal therapeutic modality for malignant head and neck tumors. Cumulative evidence indicates that unplanned radiotherapy interruption is a robust independent predictor of poorer survival outcomes across various cancers. This association is particularly more pronounced in patients with head and neck malignancies, where studies indicate that each day of interruption reduces tumor control by at least 1%. Notably, between 41.6% and 63.6% of patients with head and neck cancer experience such interruptions, primarily due to compromised nutritional status and psychological distress. Research indicates that 37.9% of cancer patients receiving radiotherapy experience weight loss during treatment. A study based on the Patient-Generated Subjective Global Assessment (PG-SGA) scale revealed that the incidence of malnutrition in patients with malignant head and neck tumors rose significantly from 24% before radiotherapy to 88% afterward. Moreover, 71.5% of patients with malignant head and neck tumors experience varying degrees of anxiety, depression, and other psychological disorders. This establishes a detrimental cycle between nutrition and psychological well-being, which may interrupt radiotherapy and impact patients' quality of life (QoL).

In China, patients often receive their RT in an outpatient setting, and nutrition clinics and psychological clinics are available only in some larger-scale hospitals, which restricts patients` access to nutritional and psychological support. Simultaneously, both oncologists and a majority of cancer patients often underestimate the importance of nutritional and psychological care, resulting in delayed interventions. These circumstances can lead to the interruption of RT, with reported rates ranging from 23.5% to 42.5% for head and neck tumors. Radiotherapy interruption directly undermines tumor control efficacy, thereby jeopardizing long-term survival outcomes in patients.

Therefore, the SHINE-MDT (comprising oncology nurse specialists, radiation oncologists, medical oncologists, dietitians, psychotherapists, and rehabilitation physicians) was established to provide nutritional, psychological, and rehabilitated support for patients with

malignant head and neck tumors throughout the entire RT period. To assess the effect of SHINE-MDT on patient outcomes, we conducted a randomized clinical trial. We hypothesized that this SHINE-MDT approach would reduce radiotherapy interruption rates and improve patients' QoL compared to usual care.

2. Objectives of the research

- **Primary Objectives:** To investigate the effect of SHINE-MDT on the rate of radiotherapy interruption.
- **Secondary objective:** To evaluate patients' QoL, nutritional status, psychological well-being, rehospitalization rate, and tumor response.
  - To investigate the impact of SHINE-MDT on patients' QoL based on the European Organization for Research and Treatment of Cancer (EORTC) Quality-of-life Questionnaire Core 30 (EORTC QLQ-C30) and Head and Neck Cancer Module (EORTC QLQ-H&N35).
  - To investigate the effect of SHINE-MDT on patients' nutritional status based on the Nutritional Risk Screening 2002 (NRS 2002) and PG-SGA.
  - To investigate the impact of SHINE-MDT care on patients' psychological status based on the Distress Thermometer (DT), Patient Health Questionnaire-9 (PHQ-9), and Hospital Anxiety and Depression Scale (HADS)
  - To investigate the impact of SHINE-MDT on the rehospitalization rate.
  - To investigate the effect of SHINE-MDT on tumor response according to RECIST version 1.1.

3. Study design, methods and procedures

3.1 Study design

A two-arm, randomized, single-center, prospective study.

3.2 Methods and Procedures

- **Patients Enrollment:**

- Inclusion criteria:

1. Male or female patients: ≥ 18 years;
2. Expected survival ≥ 6 months;
3. Pathologically confirmed malignant head and neck tumors (including nasopharyngeal carcinoma) without distant metastasis;
4. Scheduled to undergo definitive radiotherapy or postoperative adjuvant radiotherapy;
5. Systemic functional status Eastern Cooperative Oncology Group (ECOG) (Appendix 2) rating ≤ 2;
6. Adequate cognitive and reading abilities to complete the questionnaire;
7. Sign the informed consent.

- Exclusion criteria:

1. Other malignant tumors aside from head and neck malignancies (excluding treated basal cell carcinoma of the skin or in situ cervical cancer);
2. History of prior head and neck radiotherapy;
3. History of mental disorders diagnosed by a psychiatrist according to ICD-10 criteria, or history of psychotropic medication use;
4. Absolute contraindications for head and neck radiotherapy;
5. Uncontrolled systemic diseases that significantly impact the quality of life, such as poorly controlled diabetes, heart failure (NYHA classification III-IV; Appendix 4), or interstitial lung disease;

- Withdrawal Criteria:

Withdrawal criteria include, but are not limited to:

1. Patients who cancel their informed consent;
2. Patients who are non-compliant with the study protocol;

3. Patients assessed by researchers as unsuitable to continue.

- **Randomization and Blinding**

All patients were randomly assigned in a 1:1 ratio to one of two groups: the UC group or the SHINE-MDT group. The randomization process was done using a computer-generated random assignment list based on a randomized permutation block design.

Group allocation was performed using block randomization, with eligible patients being randomly assigned in a 1:1 ratio to either the UC group or the SHINE-MDT group. The statisticians designed the block size, assignment order list, and random numbers, with an equal number of patients in the UC and SHINE-MDT groups within each block. Only the statistical design personnel knew the block size (variable block size of 4 or 6). Concealment was achieved using opaque, sealed envelopes. The statistical design personnel prepared sealed envelopes with random group assignments for each participant. Sequentially, the envelopes were opened upon participant enrollment, determining the group assignment according to the allocation disclosed within the envelope.

- **Intervention Strategies**

- Anti-Cancer Treatment Regimen

All enrolled patients received radiotherapy delivered in daily fractions of 1.8 - 2.2 Gy, administered five times per week for 6 - 6.5 weeks, resulting in a total tumor dose of 60 - 72 Gy. The clinical target volume and organs at risk were delineated in accordance with consensus guidelines. Concurrent chemotherapy consisted of cisplatin 100 mg/m^2 every 3 weeks.

- Intervention Measures for the Usual Care Group

In the control group, based on the patients' questionnaire results, the radiation oncologist and risk-stratified nurses would provide dietary or psychological guidance or recommend appropriate outpatient clinics (nutrition, psychology, or rehabilitation) for patients to seek further consultation. Patients had the autonomy to decide whether or not

to schedule appointments at the recommended outpatient clinics. This reflects the routine medical practice commonly experienced by patients undergoing radiotherapy in China.

- Intervention Measures for the SHINE-MDT Group

In the experimental group, patients would be treated with SHINE-MDT in addition to standard anti-oncologic treatment. The SHINE-MDT model with a fixed-site and fixed-membership organizational framework. The team consisted of specialists from radiation oncology, medical oncology, psychology, nutrition, rehabilitation, and nursing, with each discipline maintaining ≥3 core members. At least one representative from each key department attended every meeting. This team provided direct and prompt nutritional, psychological, and rehabilitation interventions tailored to the patient's needs. All departments participated consistently, facilitating mutual learning and collaborative decision-making.

- Questionnaires Assessments

Assessments were conducted through a series of questionnaire surveys, which included the European Organization for Research and Treatment of Cancer (EORTC) Quality-of-life Questionnaire Core 30 (EORTC QLQ-C30) and Head and Neck Cancer Module (EORTC QLQ-H&N35), NRS 2002, PG-SGA, DT, PHQ-9, and HADS. The PHQ-9 and HADS were evaluated every two weeks from radiotherapy initiation through the first-month post-treatment, while the other questionnaires were assessed weekly.

- Frequency of Questionnaire Assessment

Assessments were conducted at baseline (pre-radiotherapy) and weekly from radiotherapy initiation through the first-month post-treatment, followed by evaluations at 2, 3, and 6 months post-treatment.

The end of radiotherapy: The completion of radiotherapy is the final radiotherapy day, with administration permitted within a ±1-day.

The middle of radiotherapy: The middle of radiotherapy refers to the treatment period scheduled around half of the radiotherapy schedule, within ± 1 day. Additionally, if a

patient experiences an interruption in treatment before the scheduled middle evaluation, the timing of this intermediate assessment will be delayed accordingly.

The post-treatment surveillance: Post-radiotherapy follow-up visits at 1, 2, 3, and 6 months after treatment completion refer to assessments conducted within a window of ± 3 days relative to the specified monthly intervals.

eTable 1: Frequency of Questionnaire Assessments

Test Item	Testing Time Points
Compliance with Radiotherapy	At the end of radiotherapy
EORTC QLQ-C30 Core Quality of Life Questionnaire	Baseline (pre-radiotherapy) and weekly from radiotherapy initiation through the first-month post-treatment, followed by evaluations at 2, 3, and 6 months post-treatment.
EORTC QLQ-H&N35 Head and Neck Cancer Specific Module	Baseline (pre-radiotherapy) and weekly from radiotherapy initiation through the first-month post-treatment, followed by evaluations at 2, 3, and 6 months post-treatment.
Nutritional Risk Screening Tool (NRS-2002)	Baseline (pre-radiotherapy) and weekly from radiotherapy initiation through the first-month post-treatment, followed by evaluations at 2, 3, and 6 months post-treatment.
Patient-Generated Subjective Global Assessment (PG-SGA)	Baseline (pre-radiotherapy) and weekly from radiotherapy initiation through the first-month post-treatment, followed by evaluations at 2, 3, and 6 months post-treatment.
Distress Thermometer (DT)	Baseline (pre-radiotherapy) and weekly from radiotherapy initiation through the first-month post-treatment, followed by evaluations at 2, 3, and 6 months post-treatment.
Patient Health Questionnaire-9 (PHQ-9)	Baseline (pre-radiotherapy) and once every two weeks from radiotherapy initiation through the first-month post-treatment, followed by evaluations at 2, 3, and 6 months post-treatment.
Hospital Anxiety and Depression Scale (HADS)	Baseline (pre-radiotherapy) and once every two weeks from radiotherapy initiation through the first-month post-treatment, followed by evaluations at 2, 3, and 6 months post-treatment.

- Quality control of questionnaire evaluations

To standardize questionnaire evaluations, we utilized video tutorials to assist patients in better understanding the self-rating questionnaires (EORTC QLQ-C30, EORTC QLQ-

H&N35, PG-SGA, DT, PHQ-9, HADS), enabling more accurate survey completion. For examiner-rated questionnaires (NRS 2002), nurses underwent collective training on the questionnaire content. They then conducted evaluations using standard patients to identify scoring discrepancies among different questionnaire-assessed nurses. This process enhanced understanding of the questionnaire content and reduced errors in examiner ratings.

For nutritional interventions, patients with an NRS 2002 score below 3 and a PG-SGA score below 4 were classified as having no or mild malnutrition. These patients received dietary guidance while continuing scheduled radiotherapy. For patients with a PG-SGA score of 4 or higher, different approaches were taken: those in the control group were referred to the nutrition department for consultation. In contrast, patients in the experimental group were coordinated by the risk-stratified nurses to receive individualized treatment from SHINE-MDT.

For psychological interventions, patients with mild or no psychological disorders (DT score < 4, PHQ-9 score < 10, and both HADS-A and HADS-D scores < 8) continued scheduled radiotherapy. When a patient met any of the following criteria: 1) the DT score ≥ 4, 2) the PHQ-9 score ≥ 10, 3) the HADS-A or HADS-D score ≥ 8, the risk-stratified nurses recommended that patients in the UC group go to the mental health center for psychological counseling, while the risk-stratified nurses coordinated patients in the SHINE-MDT group to receive appropriate treatment from SHINE-MDT. Patients in the control group maintained in their assigned group throughout the study.

- Research Intervention Procedure

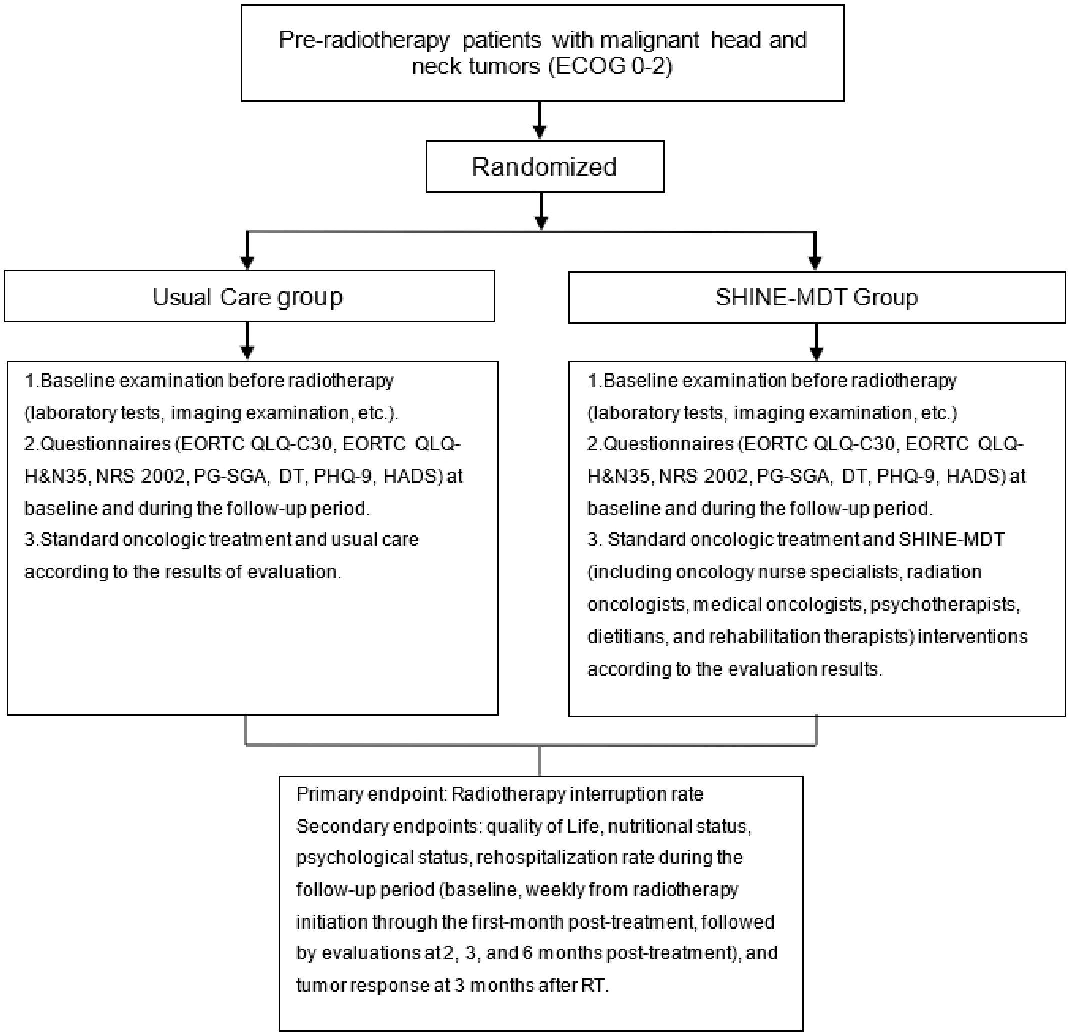

eFigure1: Research Intervention Procedure
abbreviation: SHINE-MDT: Supportive Holistic Interventions by Nurses and Experts via Multidisciplinary Team

- Nutritional and Psychological Intervention Process for the Usual Care Group

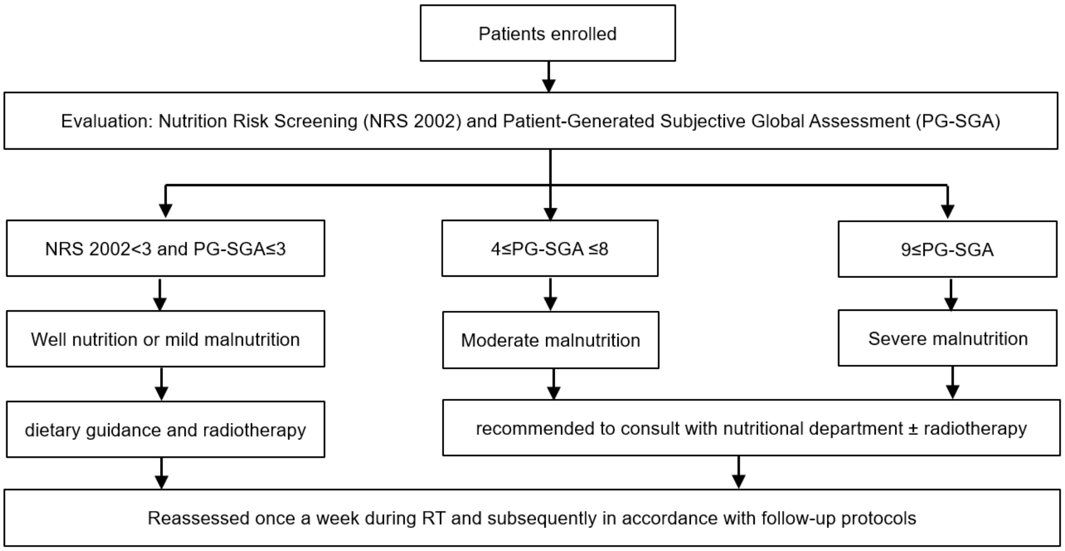

eFigure 2: Nutritional Intervention Process for the Usual Care Group

abbreviation: ONS： Oral Nutritional Supplements; PPN：Partial Parenteral Nutrition

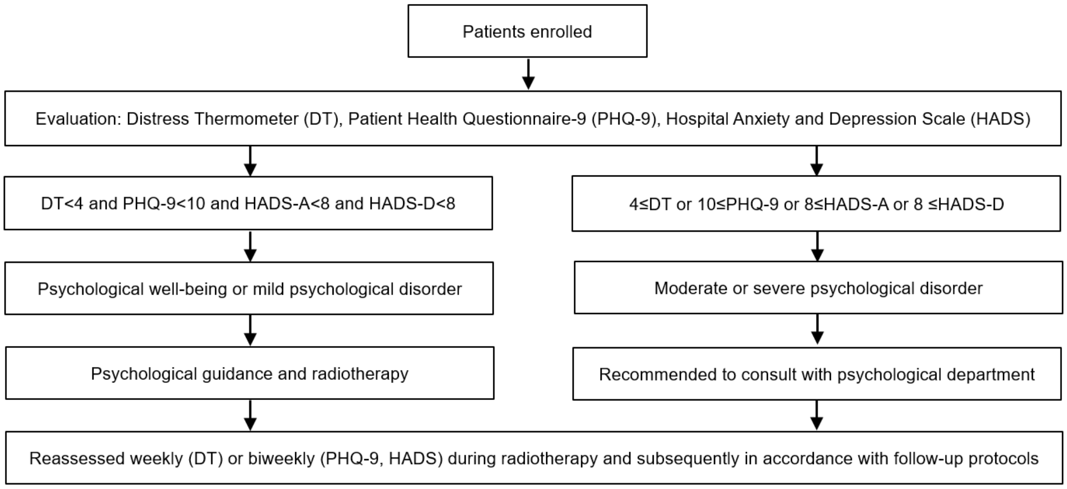

eFigure 3: Psychological Intervention Process for the Usual Care Group; HADS-A: HADS-Anxiety; HADS-D: HADS-Depression

- Nutritional and Psychological Intervention Process for the SHINE-MDT

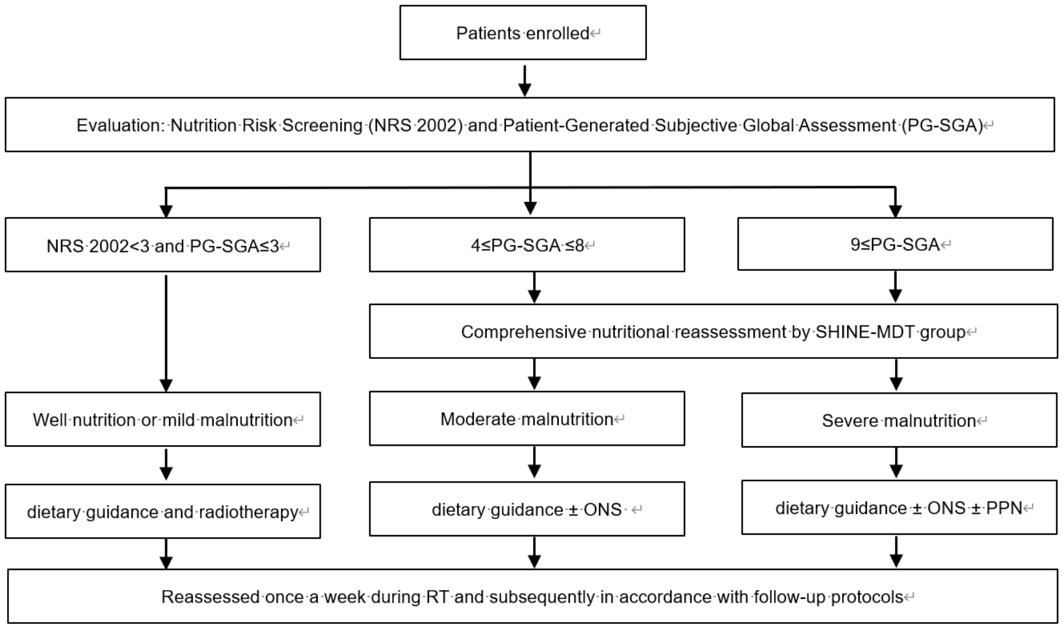

eFigure 4: Nutritional Intervention Process for the SHINE-MDT Group

abbreviation: SHINE-MDT: Supportive Holistic Interventions by Nurses and Experts via Multidisciplinary Team; ONS ： Oral Nutritional Supplements; PPN： Partial Parenteral Nutrition

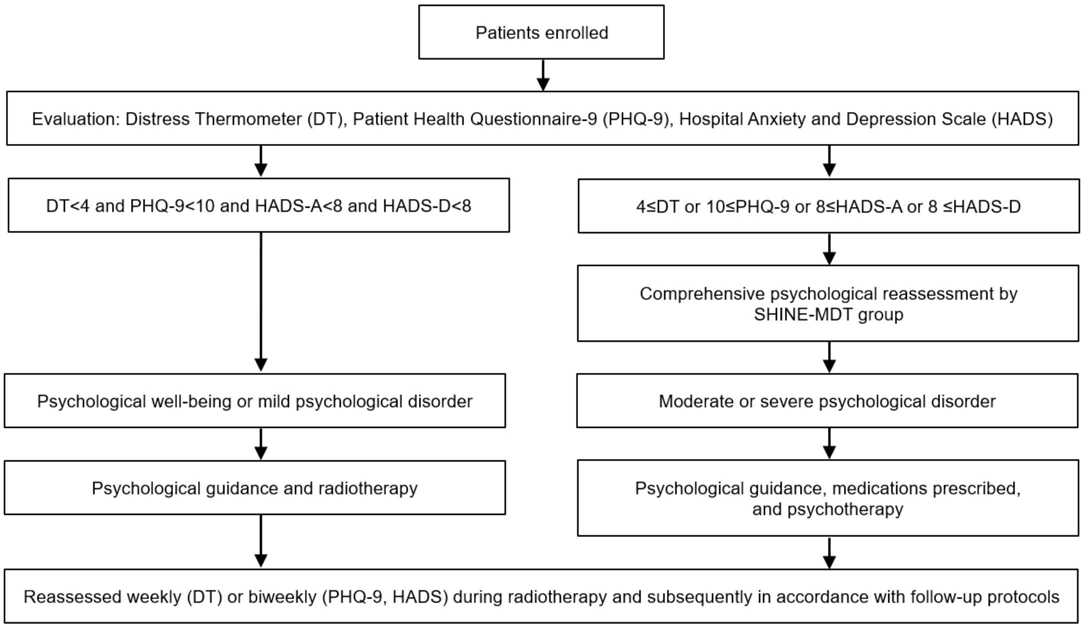

eFigure5: Psychological Intervention Process for the SHINE-MDT Group

abbreviation: SHINE-MDT: Supportive Holistic Interventions by Nurses and Experts via Multidisciplinary Team; HADS-A: HADS-Anxiety; HADS-D: HADS-Depressions

- Rehabilitation Intervention Process for the SHINE-MDT

Rehabilitation Physician: participates in MDT discussions, leads the patient's functional assessment, and formulates the rehabilitation prescription.

Specialized rehabilitation therapists then implement interventions if needed:

Speech Pathologist (SP): Addresses dysarthria, dysphonia, and dysphagia through articulation training, voice function rehabilitation, and swallowing therapy.

Occupational Therapist (OT): Manages orofacial dysfunction, ADL limitations, and cognitive deficits using adaptive equipment training, orofacial muscle strengthening, and cognitive strategy interventions.

Physical Therapist (PT): Treats motor dysfunction, pain, and lymphedema with therapeutic exercise and symptom management techniques.

Rehabilitation intervention reference for *American Cancer Society Head and Neck Cancer Survivorship Care Guideline.*

3.3 Contamination Control

- Spatial Segregation

The UC cohort received standard therapy (including radiotherapy) in the First Inpatient Department, while the SHINE-MDT cohort underwent identical protocols within a physically separated Medical Technology Building.

- Temporal Isolation

Treatment sessions for the MDT cohort were scheduled exclusively in morning blocks (08:00–13:00), with UC cohort sessions allocated to afternoon blocks (14:00–18:00). This eliminated unstructured patient interactions in shared waiting areas.

- Care Team Separation

MDT specialists (nutritional, psychological, and rehabilitation) were exclusively assigned to the intervention cohort and were not involved in UC cohort management.

4. Study assessment

4.1 Rate of radiotherapy interruption assessment

The primary endpoint was the radiotherapy interruption rate. Radiotherapy interruption was defined as actual radiotherapy days minus planned radiotherapy days greater than or equal to 5 days, regardless of the causes for interruption. The radiotherapy interruption rate was calculated as the proportion of individuals in each group who experienced such a delay. Weekends and statutory holidays were counted into the planned radiotherapy schedule.

4.2 QoL

Assessed using the EORTC QLQ-C30 and EORTC QLQ-H&N35 questionnaires.

4.3 Nutritional Status

Evaluated using NRS 2002 and PG-SGA.

4.4 Psychological Status

Assessed using the DT, PHQ-9, and HADS.

4.5 Rehospitalization

Defined as any unplanned hospital admission during the follow-up period for reasons unrelated to anti-tumor treatment.

4.6 Tumor Response

Assessed according to RECIST version 1.1 at 3 months post-RT completion, with classifications of Complete Response (CR), Partial Response (PR), Stable Disease (SD), and Progressive Disease (PD).

5. Adverse Events and Serious Adverse Events

5.1 Adverse Events (AE)

An AE is an adverse medical event that occurs in clinical study subjects after receiving treatment but is not necessarily causally related to the treatment. Adverse events also include pre-existing diseases that increase in frequency and severity during the study.

- **AEs do not encompass the following situations:**

1. Conditions, diseases, or laboratory test abnormalities pre-exist or are detected before screening and do not further deteriorate.
2. Instances where adverse medical events did not occur (such as hospitalization due to elective surgery, social reasons, and/or personal convenience)

- **Expected Adverse Events of the Study:**

  The expected adverse events in this study primarily involve those associated with radiotherapy and chemotherapy for head and neck tumors. The severity of acute radiotherapy reactions and chemotherapy-related adverse events will be evaluated using the RTOG acute radiation morbidity scoring criteria, RTOG/EORTC late radiation morbidity scoring scheme, and the NCI CTCAE Version 5.0.

5.2 Serious Adverse Events

A serious adverse event (SAE) is an adverse event that meets any of the following criteria:

1. Causing death;
2. Life-threatening;
3. Requires hospitalization or extended hospitalization;
4. Causing permanent or severe disability/incapacity;
5. Causing congenital anomalies/congenital disabilities;
6. Other significant medical events.

Significant medical events that cannot result in death, life-threatening situations, or hospitalization can also be considered SAEs related to the treatment if they may harm the subject. Based on appropriate medical diagnosis, medical or surgical intervention should be provided to prevent any of the outcomes defined above.

5.3 Classification of Adverse Events

The investigators will grade the severity of each AE according to the RTOG acute radiation morbidity scoring criteria, RTOG/EORTC late radiation morbidity scoring scheme, and the NCI CTCAE Version 5.0. If the severity of an AE is not specified in the guidelines, the investigator may assess it based on a general definition of grades 1 to 5 combined with medical judgment. The general grading of AEs is as follows:

Grade 1: Mild; asymptomatic or mild; seen only clinically or diagnostically; no treatment required.

Grade 2: Moderate; requiring minor, local, or non-invasive treatment; limitations in instrumental activities of daily living equivalent to age*.

Grade 3: Severe or medically significant but not immediately life-threatening; causing hospitalization or prolonged hospitalization; causing disability; limitations in activities of daily living**.

Grade 4: Life-threatening; requires urgent treatment.

Grade 5: Death related to AE.

* Instrumental activities of daily living include cooking, buying clothes, using the telephone, managing money, and other related activities.

** Personal activities of daily living refer to bathing, dressing, undressing, eating, grooming, medication use, etc., but do not include being bedridden.

5.4 Relevance judgment

eTable2: Adverse Drug Reaction Causality Assessment Form

Criteria	Definitely Related	Possibly Related	Possibly Unrelated	Definitely Unrelated	Unable to Determine
Reasonable Temporal Sequence	Yes	Yes	Yes	Yes	Yes
Known Treatment Reaction Type	Yes	Yes	No	No	Yes/No/Unknown
An improvement upon Causative Removal	Yes	Yes/No	Yes/No	No	Yes/No/Unknown
Recurrence upon Re-administration	Yes	Unknown	Unknown	No	Yes/No/Unknown
Alternative Explanations Possible	No	Yes/No	Yes/No	Yes	Yes/No/Unknown

6. Statistical Analysis

6.1 Determination of Sample Size

Based on literature reports and clinical experience, the radiotherapy interruption in the control group is approximately 25%, while it was expected to be 10% in the experimental group. With a significance level (α) set at 0.025 (one-sided) and a power (1-β) of 0.8, an overall predicted dropout rate of 5%, and a required sample size of 214 participants, with each group consisting of 107 participants.

6.2 Statistical Analysis Population

- **ITT (Intention-to-Treat) Set:**

This includes all randomized participants in the treatment groups, regardless of whether they completed the treatment or adhered to the protocol. The analysis is based on the initial group assignment to reflect real-world scenarios.

- **FAS (Full Analysis Set):**

This includes all participants who received the study intervention (questionnaire assessments and radiotherapy). The analysis was conducted according to the initial randomization group but may account for deviations from the protocol.

- **PPS (Per-Protocol Set):**

This includes only those participants who completed the study according to the protocol. The analysis strictly follows the randomized groups and is typically used to assess the treatment effect under ideal conditions.

References

1. Caudell JJ, Gillison ML, Maghami E, et al. NCCN Guidelines(R) Insights: Head and Neck Cancers, Version 1.2022. *J Natl Compr Canc Netw* 2022; **20**(3): 224-34.
2. Citak E, Tulek Z, Uzel O. Nutritional status in patients with head and neck cancer undergoing radiotherapy: a longitudinal study. *Support Care Cancer* 2019; **27**(1): 239-47.
3. Sroussi HY, Epstein JB, Bensadoun RJ, et al. Common oral complications of head and neck cancer radiation therapy: mucositis, infections, saliva change, fibrosis, sensory dysfunctions, dental caries, periodontal disease, and osteoradionecrosis. *Cancer Med* 2017; **6**(12): 2918-31.
4. Mallick I, Gupta SK, Ray R, et al. Predictors of weight loss during conformal radiotherapy for head and neck cancers - how important are planning target volumes? *Clin Oncol (R Coll Radiol)* 2013; **25**(9): 557-63.
5. Unsal D, Mentes B, Akmansu M, Uner A, Oguz M, Pak Y. Evaluation of nutritional status in cancer patients receiving radiotherapy: a prospective study. *Am J Clin Oncol* 2006; **29**(2): 183-8.
6. Hickok JT, Morrow GR, Roscoe JA, Mustian K, Okunieff P. Occurrence, severity, and longitudinal course of twelve common symptoms in 1129 consecutive patients during radiotherapy for cancer. *J Pain Symptom Manage* 2005; **30**(5): 433-42.
7. Jeffery DD, Art Ambrosio L, Hopkins L, Burke HB. Mental health comorbidities and cost/utilization outcomes in head and neck cancer patients. *J Psychosoc Oncol* 2019; **37**(3): 301-18.
8. Wikman A, Smedfors G, Lagergren P. Emotional distress -- a neglected topic among surgically treated oesophageal cancer patients. *Acta oncologica (Stockholm, Sweden)* 2013; **52**(8): 1783-5.
9. Yao JJ, Jin YN, Wang SY, et al. The detrimental effects of radiotherapy interruption on local control after concurrent chemoradiotherapy for advanced T-stage nasopharyngeal carcinoma: an observational, prospective analysis. *BMC Cancer* 2018; **18**(1): 740.
10. Santos M, Oliveira ESLF, Kohler HF, et al. Health-Related Quality of Life Outcomes in Head and Neck Cancer: Results From a Prospective, Real-World Data Study With Brazilian Patients Treated With Intensity Modulated Radiation Therapy, Conformal and Conventional Radiation Techniques. *International journal of radiation oncology, biology, physics* 2021; **109**(2): 485-94.
11. McCloskey SA, Jaggernauth W, Rigual NR, et al. Radiation treatment interruptions greater than one week and low hemoglobin levels (12 g/dL) are predictors of local, regional failure after definitive concurrent chemotherapy and intensity-modulated radiation therapy for squamous cell carcinoma of the head and neck. *Am J Clin Oncol* 2009; **32**(6): 587-91.
12. Yang XL, Zhou GQ, Lin L, et al. Prognostic value of radiation interruption in different periods for nasopharyngeal carcinoma patients in the intensity-modulated radiation therapy era. *Cancer Med* 2021; **10**(1): 143-55.
13. Yao JJ, Zhang F, Gao TS, et al. Survival impact of radiotherapy interruption in nasopharyngeal carcinoma in the intensity-modulated radiotherapy era: A big-data intelligence platform-based analysis. *Radiother Oncol* 2019; **132**: 178-87.
14. Young AM, Charalambous A, Owen RI, et al. Essential oncology nursing care along the cancer continuum. *The Lancet Oncology* 2020; **21**(12): e555-e63.
15. Wakefield DV, Carnell M, Dove APH, et al. Location as Destiny: Identifying Geospatial Disparities in Radiation Treatment Interruption by Neighborhood, Race, and Insurance. *International journal of radiation oncology, biology, physics* 2020; **107**(4): 815-26.
16. Yamazaki H, Nishiyama K, Tanaka E, Koizumi M, Chatani M. Radiotherapy for early glottic carcinoma (T1N0M0): results of a prospective randomized study of radiation fraction size and overall treatment time. *International journal of radiation oncology, biology, physics* 2006; **64**(1): 77-82.

17. Kwong DL, Sham JS, Chua DT, Choy DT, Au GK, Wu PM. The effect of interruptions and prolonged treatment time in radiotherapy for nasopharyngeal carcinoma. *International journal of radiation oncology, biology, physics* 1997; **39**(3): 703-10.
18. Xu C, Yang KB, Feng RJ, et al. Radiotherapy interruption due to holidays adversely affects the survival of patients with nasopharyngeal carcinoma: a joint analysis based on large-scale retrospective data and clinical trials. *Radiat Oncol* 2022; **17**(1): 36.
19. Xie J, Qi W, Cao L, et al. Predictors for Fear of Cancer Recurrence in Breast Cancer Patients Referred to Radiation Therapy During the COVID-19 Pandemic: A Multi-Center Cross-Section Survey. *Front Oncol* 2021; **11**: 650766.
20. Zimmaro LA, Sephton SE, Siwik CJ, et al. Depressive symptoms predict head and neck cancer survival: Examining plausible behavioral and biological pathways. *Cancer* 2018; **124**(5): 1053-60.
21. DiMatteo MR, Lepper HS, Croghan TW. Depression is a risk factor for noncompliance with medical treatment: meta-analysis of the effects of anxiety and depression on patient adherence. *Arch Intern Med* 2000; **160**(14): 2101-7.

Appendix 1：National Cancer Institute Common Terminology Criteria for Adverse Events (NCI CTCAE) version 5.0

For comprehensive details, please refer to the complete standard available at NCI CTCAE v5.0 or access the online information at the following NCI website: https://ctep.canCE-r.gov/protocolDevelopment/electronic_applications/ctc.htm.

Appendix 2: Eastern Cooperative Oncology Group (ECOG) - Performance Status Conversion

Score	Description
0	Fully active, able to carry on all pre-disease performances without restriction.
1	Restricted in physically strenuous activity but ambulatory and able to carry out work of a light or sedentary nature, office work
2	Ambulatory and capable of all self-care but unable to carry out any work activities; Up and about more than 50% of waking hours
3	Capable of only limited self-care, confined to bed or chair for more than 50% of waking hours
4	Completely disabled; Cannot carry on any self-care; Totally confined to bed or chair
5	Death

Appendix 3: Response Evaluation Criteria In Solid Tumours - RECIST 1.1

Note: The radiographic efficacy evaluation in this study follows the RECIST 1.1 criteria. For detailed criteria, please refer to the references: *Eisenhauer EA, Therasse P, Bogaerts J, et al. New response evaluation criteria in solid tumors: revised RECIST guideline (version 1.1). Eur. J. Cancer. 2009;45(2):228–247.*

This protocol only presents the main principles of RECIST 1.1:

1) A baseline scan is performed not more than one month before the start of treatment. The subsequent response to treatment is evaluated against this scan or the subsequent scan demonstrating the lowest sum of the target disease (nadir).

2) Target lesions are defined at baseline and must be ≥ 10 mm in longest diameter or ≥ 15 mm in the short axis if the lesion is a lymph node (although lymph nodes ≥ 10 mm are considered pathological but non-measurable). If the CT slice thickness is > 5 mm, the extranodal disease must be ≥ twice the slice thickness. Measurable disease on a chest radiograph is ≥ 20 mm, provided the lesion is "clearly defined and surrounded by aerated lung."

3) A maximum of 5 lesions may be chosen, with a maximum of 2 per organ. The sum of all the extra-nodal long-axis measurements and nodal short-axis measurements is calculated. When analyzing a follow-up scan, the same lesions are measured, and the sum of the target disease is again calculated. Measurements need not be along the same axis (as measured at baseline) but should always be the longest axis of the lesion at that point in time. It does not even have to be at the same slice position, provided the measurement is of the same lesion. However, if the initial measurements are in the axial plane, all further measurements of that lesion must remain in the axial plane. Likewise, if the initial measurements are in the coronal plane (this is acceptable although not well defined in RECIST 1.1), all further measurements of that lesion must be in the coronal plane (assumed).

4) Bone lesions with a soft tissue component ≥ 10 mm can be designated as target lesions. Sclerotic bone lesions can not be used. Although solid lesions should be used in preference, cystic lesions may be used, provided they represent the disease being studied.

5) Evaluation of target lesions

   ① Complete Response (CR): Disappearance of all target lesions. Any pathological lymph nodes (whether target or non-target) must have a reduction in the short axis to

<10 mm.

② Partial Response (PR): At least a 30% decrease in the sum of diameters of target lesions, taking as reference the baseline sum diameters.

③ Progressive Disease (PD): At least a 20% increase in the sum of diameters of target lesions, taking as reference the smallest sum in the study (this includes the baseline sum if that is the smallest in the study). In addition to the relative increase of 20%, the sum must also demonstrate an absolute increase of at least 5 mm. (Note: the appearance of one or more new lesions is also considered progression).

④ Stable Disease (SD): Neither sufficient shrinkage to qualify for PR nor sufficient increase to qualify for PD, taking as reference the smallest sum diameters while on study.

6) If a lesion reappears after disappearing in a patient with complete response, progressive disease is declared. However, if such a lesion behaves in this manner in a patient with stable disease or partial response, it is the change in sum of target disease that defines the response or progression.

7) PET-CT and MR may be used. The main specification is that imaging protocols are consistent throughout the trial. Therefore, contrast timings, MR sequences, and planes should remain the same.

Table 3-1. Timepoint response: patients with target (+/–non-target) disease

Target lesions	Non-target lesions	New lesions	Overall response
CR	CR	No	CR
CR	Non-CR/non-PD	No	PR
CR	Not evaluated	No	PR
PR	Non-PD or not all evaluated	No	PR
SD	Non-PD or not all evaluated	No	SD
Not all evaluated	Non-PD	No	NE
PD	Any	Yes or No	PD
Any	PD	Yes or No	PD

Target lesions	Non-target lesions	New lesions	Overall response
Any	Any	Yes	PD

CR = complete response, PR = partial response, SD = stable disease, PD = progressive disease, and NE = inevaluable.

Table 3-2. Timepoint response: patients with non-target disease only

Non-target lesions	New lesions	Overall response
CR	No	CR
Non-CR/non-PD	No	Non-CR/non-PD[a]
Not all evaluated	No	NE
Unequivocal PD	Yes or No	PD
Any	Yes	PD

CR = complete response, PD = progressive disease, and NE = inevaluable.

A ‘Non-CR/non-PD’ is preferred over ‘stable disease’ for non-target disease since SD is increasingly used as endpoint for assessment of efficacy in some trials so to assign this category when no lesions can be measured is not advised.

Table 3-3. The best overall response is when confirmation of CR and PR is required.

Overall response<br>First-time point	Overall response<br>Subsequent time point	Best overall response
CR	CR	CR
CR	PR	SD, PD or PR[a]
CR	SD	SD provided minimum criteria for SD duration met; otherwise, PD
CR	PD	SD provided minimum criteria for SD duration met; otherwise, PD
CR	NE	SD provided minimum criteria for SD duration met; otherwise, PD
PR	CR	PR
PR	PR	PR
PR	SD	SD
PR	PD	SD provided minimum criteria for SD duration met; otherwise, PD
PR	NE	SD provided minimum criteria for SD duration met; otherwise, NE
NE	NE	NE

CR = complete response, PR = partial response, SD = stable disease, PD = progressive disease, and NE = inevaluable.

a If a CR is truly met at the first time point, then any disease seen at a subsequent time point, even a disease meeting PR criteria relative to baseline, makes the disease PD at that point (since the disease must have reappeared after CR). The best response would depend on whether the minimum duration for SD was met. However, sometimes 'CR' may be claimed when subsequent scans suggest small lesions were likely still present and, in fact, the patient had PR, not CR, at the first time point. Under these circumstances, the original CR should be changed to PR, and the best response should be PR.

Appendix 4: New York Heart Association (NYHA) Functional Classification

Class	Description
0	No heart disease
I	No limitation on physical activity. Ordinary physical activity does not cause undue fatigue, palpitation (feeling heartbeats), or dyspnea (shortness of breath)
II	Slight limitation of physical activity. Comfortable at rest, but ordinary physical activity results in fatigue, palpitation, or dyspnea
III	Marked limitation of physical activity. Comfortable at rest, but less than ordinary activity causes fatigue, palpitation, or dyspnea
IV	Unable to carry out any physical activity without discomfort. Symptoms of cardiac insufficiency at rest. If any physical activity is undertaken, discomfort is increased.

Appendix 5: Case Report Form (English version)

Patient General Information Questionnaire

No.: Date:

Dear Patient,

Hello! Thank you for your trust in our hospital. To better understand your health condition and provide you with more comprehensive healthcare management services, we have designed this questionnaire. We kindly ask for your cooperation in completing it. The information you provide will be used solely for research purposes, and your personal privacy will be strictly protected.

Please read each question carefully and answer truthfully based on your actual situation. Your responses are crucial to the conclusions of our study. We sincerely appreciate your cooperation!

Thank you very much!

I. General Information

1. Gender: ① Male ② Female

2. Ethnicity: ① Han ② Other (please specify): _______

3. Age: _______ years

4. Education Level:

①Primary school or below ② Junior high school

③ High school / Technical secondary school / College ④ Bachelor's degree or above

5. Marital Status:

① Single ② Married ③ Divorced or widowed

6. Occupation Before Illness:

① Farmer ② Worker ③ Civil servant ④ Self-employed ⑤ Unemployed

⑥ Retired ⑦ Other (please specify): _______

7. Employment Status After Illness:

① Employed ② Unemployed ③ Retired

8. Monthly Household Income per Capita (RMB):

① <1,000 ② 1,000–2,999 ③ 3,000–4,999 ④ ≥5,000

9. Medical Payment Method:

① Self-paid ② Commercial insurance ③ Urban employee medical insurance

④ Urban resident medical insurance ⑤Other (please specify): _______

10. Primary Caregiver:

① Spouse ② Children ③ Parents

④ Other (please specify): _______

II. Disease-Related Information

1. Disease Site:

① Nasopharynx ② Nasal cavity & paranasal sinuses ③ Larynx & hypopharynx ④ Oral cavity ⑤ Major salivary glands ⑥ Oropharynx ⑦ Other (please specify): _______

2. Stage:

① Stage I ② Stage II ③ Stage III ④ Stage IV

3. ECOG Performance Status:

① 0 ② 1 ③ 2

4. Current Weight (kg): _______ (Assessed weekly)

5. BMI (kg/m²): _______

Scale I: QLQ-C30 Quality of Life Questionnaire

EORTC QLQ-C30 (version 3)

We are interested in some things about you and your health. Please answer all of the questions yourself by circling the number that best applies to you. There are no "right" or "wrong" answers. The information that you provide will remain strictly confidential.

Today's date (Day, Month, Year): |_|_|_|_|_|_|_|_|

	Not at All	A Little	Quite a Bit	Very Much
1. Do you have any trouble doing strenuous activities, like carrying a heavy shopping bag or a suitcase?	1	2	3	4
2. Do you have any trouble taking a long walk?	1	2	3	4
3. Do you have any trouble taking a short walk outside of the house?	1	2	3	4
4. Do you need to stay in bed or a chair during the day?	1	2	3	4
5. Do you need help with eating, dressing, washing yourself or using the toilet?	1	2	3	4

**During the past week:**	Not at All	A Little	Quite a Bit	Very Much
6. Were you limited in doing either your work or other daily activities?	1	2	3	4
7. Were you limited in pursuing your hobbies or other leisure time activities?	1	2	3	4
8. Were you short of breath?	1	2	3	4
9. Have you had pain?	1	2	3	4
10. Did you need to rest?	1	2	3	4
11. Have you had trouble sleeping?	1	2	3	4
12. Have you felt weak?	1	2	3	4
13. Have you lacked appetite?	1	2	3	4
14. Have you felt nauseated?	1	2	3	4
15. Have you vomited?	1	2	3	4
16. Have you been constipated?	1	2	3	4

Please go on to the next page

**During the past week:**	**Not at All**	**A Little**	**Quite a Bit**	**Very Much**
17. Have you had diarrhea?	1	2	3	4
18. Were you tired?	1	2	3	4
19. Did pain interfere with your daily activities?	1	2	3	4
20. Have you had difficulty in concentrating on things, like reading a newspaper or watching television?	1	2	3	4
21. Did you feel tense?	1	2	3	4
22. Did you worry?	1	2	3	4
23. Did you feel irritable?	1	2	3	4
24. Did you feel depressed?	1	2	3	4
25. Have you had difficulty remembering things?	1	2	3	4
26. Has your physical condition or medical treatment interfered with your family life?	1	2	3	4
27. Has your physical condition or medical treatment interfered with your social activities?	1	2	3	4
28. Has your physical condition or medical treatment caused you financial difficulties?	1	2	3	4

For the following questions please circle the number between 1 and 7 that best applies to you

29. How would you rate your overall health during the past week?

1	2	3	4	5	6	7
Very poor						Excellent

30. How would you rate your overall quality of life during the past week?

1	2	3	4	5	6	7
Very poor						Excellent

© Copyright 1995 EORTC Quality of Life Group. All rights reserved. Version 3.0

EORTC QLQ - H&N35

Patients sometimes report that they have the following symptoms or problems. Please indicate the extent to which you have experienced these symptoms or problems during the past week. Please answer by circling the number that best applies to you.

During the past week:	Not at all	A little	Quite a bit	Very much
31. Have you had pain in your mouth?	1	2	3	4
32. Have you had pain in your jaw?	1	2	3	4
33. Have you had soreness in your mouth?	1	2	3	4
34. Have you had a painful throat?	1	2	3	4
35. Have you had problems swallowing liquids?	1	2	3	4
36. Have you had problems swallowing pureed food?	1	2	3	4
37. Have you had problems swallowing solid food?	1	2	3	4
38. Have you choked when swallowing?	1	2	3	4
39. Have you had problems with your teeth?	1	2	3	4
40. Have you had problems opening your mouth wide?	1	2	3	4
41. Have you had a dry mouth?	1	2	3	4
42. Have you had sticky saliva?	1	2	3	4
43. Have you had problems with your sense of smell?	1	2	3	4
44. Have you had problems with your sense of taste?	1	2	3	4
45. Have you coughed?	1	2	3	4
46. Have you been hoarse?	1	2	3	4
47. Have you felt ill?	1	2	3	4
48. Has your appearance bothered you?	1	2	3	4

Please go on to the next page

**During the past week:**	**Not at all**	**A little**	**Quite a bit**	**Very much**
49. Have you had trouble eating?	1	2	3	4
50. Have you had trouble eating in front of your family?	1	2	3	4
51. Have you had trouble eating in front of other people?	1	2	3	4
52. Have you had trouble enjoying your meals?	1	2	3	4
53. Have you had trouble talking to other people?	1	2	3	4
54. Have you had trouble talking on the telephone?	1	2	3	4
55. Have you had trouble having social contact with your family?	1	2	3	4
56. Have you had trouble having social contact with friends?	1	2	3	4
57. Have you had trouble going out in public?	1	2	3	4
58. Have you had trouble having physical contact with family or friends?	1	2	3	4
59. Have you felt less interest in sex?	1	2	3	4
60. Have you felt less sexual enjoyment?	1	2	3	4

**During the past week:**	**No**	**Yes**
61. Have you used pain-killers?	1	2
62. Have you taken any nutritional supplements (excluding vitamins)?	1	2
63. Have you used a feeding tube?	1	2
64. Have you lost weight?	1	2
65. Have you gained weight?	1	2

© Copyright QLQ-H&N35 1994 EORTC Quality of Life Group, version 1. All rights reserved

Scale II: Nutrition Risk Screening (NRS-2002)

Category	Content	Score
**1. Disease Severity Score**	Pelvic fracture or chronic disease patients with any of the following: cirrhosis, COPD, long-term hemodialysis, diabetes, cancer.	1
	Major abdominal surgery, stroke, severe pneumonia, hematologic malignancies.	2
	Traumatic brain injury, bone marrow suppression, ICU patients (APACHE >10).	3
	**Subtotal**	
**2.Impaired Nutritional Status Score**	Normal nutritional status, BMI ≥18.5, no weight change in the past 1–3 months, no change in food intake in the past week.	0
	Weight loss >5% in the past 3 months or 20%–50% reduction in food intake (compared to requirements) in the past week.	1
	Weight loss >5% in the past 2 months or BMI 18.5–20.5 or 50%–75% reduction in food intake (compared to requirements) in the past week.	2
	Weight loss >5% in the past month (or >15% in 3 months) or BMI <18.5 (or serum albumin <35 g/L) or 70%–100% reduction in food intake (compared to requirements) in the past week.	3
	**Subtotal**	
**3. Age Score**	Age ≥70 years	1
**Total Nutritional Risk Screening Score**		

Scale III: Scored Patient-Generated Subjective Global Assessment (PG-SGA)

Scored Patient-Generated Subjective Global Assessment (PG-SGA)

History: Boxes 1 - 4 are designed to be completed by the patient.
[Boxes 1-4 are referred to as the PG-SGA Short Form (SF)]

Patient Identification Information

1. Weight *(See Worksheet 1)*

In summary of my current and recent weight:

I currently weigh about _____kg
I am about _____ cm tall

One month ago I weighed about _____ kg
Six months ago I weighed about _____ kg

During the past two weeks my weight has:
☐ decreased (1) ☐ not changed (0) ☐ increased (0)

Box 1 ☐

2. Food intake: As compared to my normal intake, I would rate my food intake during the past month as
- ☐ unchanged (0)
- ☐ more than usual (0)
- ☐ less than usual (1)

I am now taking
- ☐ *normal food* but less than normal amount (1)
- ☐ little solid food (2)
- ☐ only liquids (3)
- ☐ only nutritional supplements (3)
- ☐ very little of anything (4)
- ☐ only tube feedings or only nutrition by vein (0)

Box 2 ☐

3. Symptoms: I have had the following problems that have kept me from eating enough during the past two weeks (check all that apply)
- ☐ no problems eating (0)
- ☐ no appetite, just did not feel like eating (3)
- ☐ vomiting (3)
- ☐ nausea (1)
- ☐ diarrhea (3)
- ☐ constipation (1)
- ☐ dry mouth (1)
- ☐ mouth sores (2)
- ☐ smells bother me (1)
- ☐ things taste funny or have no taste (1)
- ☐ feel full quickly (1)
- ☐ problems swallowing (2)
- ☐ fatigue (1)
- ☐ pain; where? (3) _________________
- ☐ other (1)** _____________________

**Examples: depression, money, or dental problems

Box 3 ☐

4. Activities and Function:
Over the past month, I would generally rate my activity as:
- ☐ normal with no limitations (0)
- ☐ not my normal self, but able to be up and about with fairly normal activities (1)
- ☐ not feeling up to most things, but in bed or chair less than half the day (2)
- ☐ able to do little activity and spend most of the day in bed or chair (3)
- ☐ pretty much bed ridden, rarely out of bed (3)

Box 4 ☐

Additive Score of Boxes 1-4 (See Side 1) ☐ **A**

Worksheet 1 – Scoring Weight Loss

To determine score, use 1-month weight data if available. Use 6-month data only if there is no 1-month weight data. Use points below to score weight change and add one extra point if patient has lost weight during the past 2 weeks. Enter total point score in Box 1 of PG-SGA.

Weight loss in 1 month	Points	Weight loss in 6 months
10% or greater	4	20% or greater
5-9.9%	3	10- 19.9%
3-4.9%	2	6- 9.9%
2-2.9%	1	2- 5.9%
0-1.9%	0	0- 1.9%

Numerical score from Worksheet 1 ☐

5. Worksheet 2 – Disease and its relation to nutritional requirements:

Score is derived by adding 1 point for each of the following conditions:
- ☐ Cancer
- ☐ Presence of decubitus, open wound or fistula
- ☐ AIDS
- ☐ Presence of trauma
- ☐ Pulmonary or cardiac cachexia
- ☐ Age greater than 65
- ☐ Chronic renal insufficiency

Other relevant diagnoses (specify) ___________________________________
Primary disease staging (circle if known or appropriate) I II III IV Other ______

Numerical score from Worksheet 2 ☐ **B**

6. Worksheet 3 – Metabolic Demand

Score for metabolic stress is determined by a number of variables known to increase protein & caloric needs. **Note:** Score fever intensity or duration, whichever is greater. The score is additive so that a patient who has a fever of 38.8 °C (3 points) for < 72 hrs (1 point) and who is on 10 mg of prednisone chronically (2 points) would have an additive score for this section of 5 points.

Stress	none (0)	low (1)	moderate (2)	high (3)
**Fever**	no fever	> 37.2 and < 38.3	≥ 38.3 and < 38.8	≥ 38.8 °C
**Fever duration**	no fever	< 72 hours	72 hours	> 72 hours
**Corticosteroids**	no corticosteroids	low dose (< 10 mg prednisone equivalents/day)	moderate dose (≥ 10 and < 30 mg prednisone equivalents/day)	high dose (≥ 30 mg prednisone equivalents/day)

Numerical score from Worksheet 3 ☐ **C**

7. Worksheet 4 – Physical Exam

Exam includes a subjective evaluation of 3 aspects of body composition: fat, muscle, & fluid. Since this is subjective, each aspect of the exam is rated for degree. Muscle deficit/loss impacts point score more than fat deficit/loss. Definition of categories: 0 = no abnormality, 1+ = mild, 2+ = moderate, 3+ = severe. Rating in these categories is *not* additive but are used to clinically assess the degree of deficit (or presence of excess fluid).

Muscle Status				
temples (temporalis muscle)	0	1+	2+	3+
clavicles (pectoralis & deltoids)	0	1+	2+	3+
shoulders (deltoids)	0	1+	2+	3+
interosseous muscles	0	1+	2+	3+
scapula (latissimus dorsi, trapezius, deltoids)	0	1+	2+	3+
thigh (quadriceps)	0	1+	2+	3+
calf (gastrocnemius)	0	1+	2+	3+
**Global muscle status rating**	**0**	**1+**	**2+**	**3+**

Fat Stores				
orbital fat pads	0	1+	2+	3+
triceps skin fold	0	1+	2+	3+
fat overlying lower ribs	0	1+	2+	3+
**Global fat deficit rating**	**0**	**1+**	**2+**	**3+**
**Fluid status**				
ankle edema	0	1+	2+	3+
sacral edema	0	1+	2+	3+
ascites	0	1+	2+	3+
**Global fluid status rating**	**0**	**1+**	**2+**	**3+**

Point score for the physical exam is determined by the overall subjective rating of the total body deficit.

No deficit	score = 0 points
Mild deficit	score = 1 point
Moderate deficit	score = 2 points
Severe deficit	score = 3 points

Again, muscle deficit/loss takes precedence over fat loss or fluid excess.

Numerical Score for Worksheet 4 ☐ **D**

Total PG-SGA Score (Total numerical score of A+B+C+D) ☐

Clinician Signature _____________________________________RD RN PA MD DO Other ________ Date _______________

Global PG-SGA Category Rating (Stage A, Stage B or Stage C) ☐

Scale IV: Patient Health Questionnaire-9 (PHQ-9) Depression Screening Scale

PATIENT HEALTH QUESTIONNAIRE (PHQ-9)

ID #: ______________________ **DATE:** ______________

Over the last *2 weeks,* how often have you been bothered by any of the following problems? *(use "✓" to indicate your answer)*	Not at all	Several days	More than half the days	Nearly every day
**1.** Little interest or pleasure in doing things	0	1	2	3
**2.** Feeling down, depressed, or hopeless	0	1	2	3
**3.** Trouble falling or staying asleep, or sleeping too much	0	1	2	3
**4.** Feeling tired or having little energy	0	1	2	3
**5.** Poor appetite or overeating	0	1	2	3
**6.** Feeling bad about yourself—or that you are a failure or have let yourself or your family down	0	1	2	3
**7.** Trouble concentrating on things, such as reading the newspaper or watching television	0	1	2	3
**8.** Moving or speaking so slowly that other people could have noticed. Or the opposite — being so figety or restless that you have been moving around a lot more than usual	0	1	2	3
**9.** Thoughts that you would be better off dead, or of hurting yourself	0	1	2	3
	add columns	____ +	____ +	____

(Healthcare professional: For interpretation of TOTAL, please refer to accompanying scoring card). TOTAL: ______

Scale V: Distress Thermometer (DT) Psychological Screening Tool

Distress is an unpleasant experience of a mental, physical, social, or spiritual nature. It can affect the way you think, feel, or act. Distress may make it harder to cope with having cancer, its symptoms, or its treatment.

Instructions: Please circle the number (0–10) that best describes how much distress you have been experiencing in the past week, including today.

Extreme distress

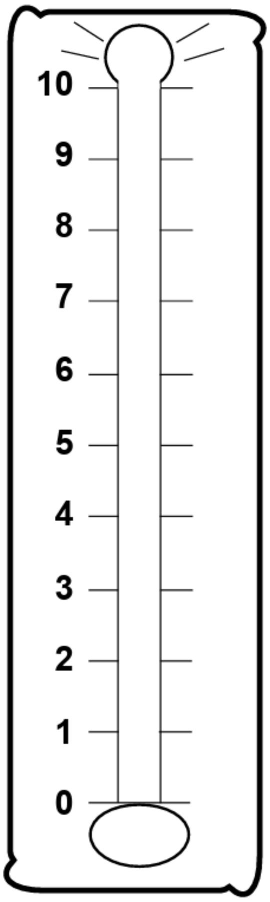

No distress

Scale 6: Hospital Anxiety and Depression Scale (HADS)

Instructions: Emotions play a significant role in most illnesses. If healthcare providers understand your emotional state, they can offer better support. Please read each statement below and check (√) the response that best describes how you have felt over the past month. Do not overthink your answers—your immediate response is likely more accurate.

1. I feel tense or wound up: (   )
   ① Almost all the time
   ② Most of the time
   ③ Sometimes
   ④ Not at all
2. I still enjoy the things I used to enjoy: (   )
   ① Definitely as much
   ② Not quite as much
   ③ Only a little
   ④ Hardly at all
3. I get a sort of frightened feeling, as if something awful might happen: (   )
   ① Very definitely and quite badly
   ② Yes, but not too badly
   ③ A little, but it doesn't worry me
   ④ Not at all
4. I can laugh and see the funny side of things: (   )
   ① As much as I always could
   ② Not quite so much now
   ③ Definitely not so much now
   ④ Not at all
5. Worrying thoughts go through my mind: (   )
   ① A great deal of the time
   ② A lot of the time
   ③ From time to time, but not too often
   ④ Only occasionally
6. I feel cheerful: (   )
   ① Not at all
   ② Not often
   ③ Sometimes
   ④ Most of the time
7. I can sit at ease and feel relaxed: (   )
   ① Definitely
   ② Usually
   ③ Not often
   ④ Not at all
8. I have lost interest in my appearance: (   )
   ① Definitely
   ② I don't take as much care as I should

③ I may not take quite as much care
④ I take just as much care as ever

9. I feel restless, as if I have to be on the move: ( )
① Very much indeed
② Quite a lot
③ Not very much
④ Not at all

10. I look forward with enjoyment to things: ( )
① As much as I ever did
② Rather less than I used to
③ Definitely less than I used to
④ Hardly at all

11. I get sudden feelings of panic: ( )
① Very often indeed
② Quite often
③ Not very often
④ Not at all

12. I feel as if I'm slowing down: ( )
① Nearly all the time
② Very often
③ Sometimes
④ Not at all

13. I have a feeling of dread, as if something bad is about to happen to my body: ( )
① Not at all
② Sometimes
③ Very often
④ Most of the time

14. I can enjoy a good book, radio, or TV program: ( )
① Often
② Sometimes
③ Not often
⑤ Very seldom

Appendix 6 Informed consent (English version)

Informed Consent Form for the Randomized Controlled Clinical Study on the Development and Evaluation of a Comprehensive Multidisciplinary Care Model for Head and Neck Cancer Patients Undergoing Radiotherapy

Dear Participant,

We invite you to participate in the research project titled **"Randomized Controlled Clinical Study on the Development and Evaluation of a Comprehensive Multidisciplinary Care Model for Head and Neck Cancer Patients Undergoing Radiotherapy,"** approved by West China Hospital, Sichuan University. An estimated 214 participants will voluntarily join this study. The research has been reviewed and approved by the Biomedical Ethics Committee of West China Hospital, Sichuan University.

1. Why is this study being conducted?

Radiotherapy is one of the primary treatments for head and neck tumors (including nasopharyngeal carcinoma). Side effects such as reduced taste sensitivity, radiation-induced oral mucositis, and dry mouth can lead to decreased nutrient intake, worsening nutritional status, or malnutrition. Studies show that patients undergoing definitive radiotherapy for head and neck malignancies lose an average of 3.8% of their body weight during treatment, with 56 - 88% experiencing a weight loss of >5%. The incidence of radiotherapy-related adverse reactions exceeds 90%, with grade 3 or higher adverse events potentially leading to treatment interruptions. Reports indicate that radiotherapy interruptions occur in 23.5%–42.5% of head and neck cancer (including nasopharyngeal carcinoma) cases. Treatment interruptions not only affect therapeutic efficacy and long-term survival but also significantly reduce patients' quality of life.

Head and neck cancer patients also experience varying degrees of psychological distress, such as anxiety, depression, and social dysfunction, which can further exacerbate malnutrition. Conversely, concerns about malnutrition may worsen emotional distress, creating a vicious cycle that negatively impacts quality of life.

Rehabilitation is a crucial component of head and neck cancer treatment, as it helps patients restore physical function, improve quality of life, and enhance treatment adherence. Early intervention, such as neck and shoulder exercises, can effectively prevent muscle atrophy and mobility limitations caused by radiotherapy and lymph node dissection. Furthermore, because radiation-induced muscle fibrosis can impair essential functions like swallowing and mouth opening, rehabilitation is key to sustaining a patient's treatment persistence and quality of life. Studies demonstrate that this training not only improves physical function but also alleviates fatigue, which leads to a significant overall enhancement of a patient's well-being.

Therefore, this randomized controlled clinical study aims to develop a **comprehensive multidisciplinary care model** integrating **nutrition, psychological support, and rehabilitation** for head and neck cancer patients undergoing radiotherapy. The goal is to reduce treatment interruption rates and improve patients' quality of life.

2. What will you need to do if you participate?

To see if you are eligible for the study, your doctor will first review your medical history, perform a physical exam, and relevant tests. These tests include ECOG scoring, blood work, and checks on your liver, kidney, and electrolytes.

If you are eligible, you can choose to join the study by signing this consent form. If you do not wish to join, you will still receive standard treatment as your doctor have planned. If you do join, you will undergo the study's interventions and related follow-ups.

Nurses will meet with you at the start of the study (before radiotherapy), once a week during radiotherapy, and then again at 1, 2, 3, and 6 months after radiotherapy. During these times, you will be asked to fill out questionnaires and follow the medical advice given to you.

3. Who should not participate?

You are ineligible if you meet any of the following criteria:

1. You have another type of cancer, except for a successfully treated basal cell carcinoma or cervical carcinoma in situ.
2. You have previously received radiotherapy.
3. You have a history of a psychiatric condition or a cognitive impairment.
4. You have a health condition that would prevent you from safely receiving head and neck radiotherapy.
5. You have an uncontrolled systemic disease that significantly affects your quality of life, such as poorly managed diabetes, NYHA Class III-IV heart failure, or interstitial lung disease.

4. What are the potential risks?

This study involves no experimental drugs or new technologies; it focuses on developing a **multidisciplinary care model**, with benefits outweighing risks.

Radiotherapy or chemoradiotherapy for head and neck cancer (including nasopharyngeal carcinoma) may cause unavoidable side effects. Your doctor will proactively manage known adverse reactions and adjust treatment as needed.

If you experience discomfort, disease progression, or unexpected events during the study, notify your doctor immediately for evaluation and appropriate care.

Follow-ups and tests may require time and inconvenience.

5. What are the potential benefits?

Participation may improve your condition and help determine safer, more effective treatments for future patients.

(1) Experienced doctors will oversee your care, address concerns, and provide timely medical support. The study follows a detailed, ethics-approved protocol to protect participants' rights.

(2) The care model may alleviate symptoms, individual outcomes vary. Participants will receive timely scale-based assessments and follow-ups, with corresponding feedback provided based on the results. Participants assigned to the control group will receive the current standard medical care. Participants assigned to the intervention group will receive comprehensive care from an MDT. The findings from this study will help inform the development of future clinical practice guidelines.

6. Are there any costs involved?

No additional costs are required for participation.

7. Is your personal information confidential?

Your data will be stored at West China Hospital, accessible only to researchers, regulatory authorities, and the ethics committee. Published results will not disclose your identity. We uphold strict confidentiality within legal limits.

8. Is participation mandatory?

Participation is **entirely voluntary**. You may decline or withdraw anytime without penalty, affecting neither your medical care nor rights. If withdrawing, inform your doctor for proper follow-up.

Subject Statement: I have read the above information regarding this study. The researchers have fully explained and described to me the purpose, procedures, potential risks, and benefits of participating in this research, and all my questions have been answered. I voluntarily agree to participate in this study.

I agree ☐ or refuse ☐ to the use of my research data and biological specimens for other research beyond this study.

Subject's Printed Name: ________________

Subject's Signature: _______________ Date:______Year___Month___Day

Subject's Contact Phone: ______________ Mobile: ______________

Legal Representative's Printed Name: ________________ (if applicable)

Relationship to Subject: ________________

Legal Representative's Signature: ________________Date:______Year___Month___Day

Reason for Legal Representative's Signature: ____________________________________

Witness's Printed Name: ________________ (if applicable)

Witness's Signature: ________________ Date:______Year___Month___Day

Reason for Witness's Signature: ____

Physician's Statement: I have explained the details of this study to the above-named volunteer and provided him/her with a signed original copy of the informed consent form. I confirm that the study has been thoroughly explained to the subject, particularly regarding ethical principles and requirements such as potential risks/benefits, free provisions/compensation, harm/compensation, voluntariness, and confidentiality.

Physician's Signature: _______________ Date:______Year___Month___Day

Physician's Contact Phone: ______________

Biomedical Ethics Committee West China Hospital, Sichuan University

Tel: 028-85422654, 028-85423237
