# Supplementary material for: Multidisciplinary Team Support for Patients With Head and Neck Cancer Receiving Radiotherapy: A Randomized Clinical Trial
Source: JAMA Netw Open. 2025 Dec 15;8(12):e2547590. doi: 10.1001/jamanetworkopen.2025.47590 (PMC12706684; doi:10.1001/jamanetworkopen.2025.47590)
Supplement: Supplement 2. — eMethods eFigure 1. Overview of the Study Workflow eFigure 2. Schematic Representation of the SHINE-MDT Intervention Flow eFigure 3. Quality of Life (EORTC QLQ-C30/QLQ-H&N35) Assessment Scores in UC and SHINE-MDT Groups Over Time eTable 1. Comparison of Radiotherapy Dose Parameters Between Treatment Groups eTable 2. Comparison of Consultation Recommendations and Completion Between UC Group and SHINE-MDT Group eTable 3. Reasons for Radiotherapy Interruptions—UC Group vs SHINE-MDT Group eTable 4. Longitudinal Changes in Quality of Life, Nutrition Status, and Psychological Status During Radiotherapy in Patients With Malignant Head and Neck Tumor eTable 5. Cronbach α Values Across Assessment Time Points eTable 6. Comparison of Weight Between UC and SHINE-MDT Groups During and After Radiotherapy eTable 7. Reasons for Rehospitalization Events—UC Group vs SHINE-MDT Group eTable 8. Tumor Response Between UC Group and SHINE-MDT Group [file jamanetwopen-e2547590-s002.pdf]

Supplementary Online Content

Pei Y, Wang J, Li J, et al. Multidisciplinary team support for patients with head and neck cancer receiving radiotherapy: a randomized clinical trial. *JAMA Netw Open*. 2025;8(12):e2547590. doi:10.1001/jamanetworkopen.2025.47590

eMethods
eFigure 1. Overview of the Study Workflow
eFigure 2. Schematic Representation of the SHINE-MDT Intervention Flow
eFigure 3. Quality of Life (EORTC QLQ-C30/QLQ-H&N35) Assessment Scores in UC and SHINE-MDT Groups Over Time
eTable 1. Comparison of Radiotherapy Dose Parameters Between Treatment Groups
eTable 2. Comparison of Consultation Recommendations and Completion Between UC Group and SHINE-MDT Group
eTable 3. Reasons for Radiotherapy Interruptions—UC Group vs SHINE-MDT Group
eTable 4. Longitudinal Changes in Quality of Life, Nutrition Status, and Psychological Status During Radiotherapy in Patients With Malignant Head and Neck Tumor
eTable 5. Cronbach α Values Across Assessment Time Points
eTable 6. Comparison of Weight Between UC and SHINE-MDT Groups During and After Radiotherapy
eTable 7. Reasons for Rehospitalization Events—UC Group vs SHINE-MDT Group
eTable 8. Tumor Response Between UC Group and SHINE-MDT Group

This supplementary material has been provided by the authors to give readers additional information about their work.

© 2025 Pei Y et al. *JAMA Network Open*.

eMETHODS

In this supplement, we provide detailed descriptions of the methods.

1.1 Determination of Sample Size

Based on literature evidence and clinical experience, the radiotherapy interruption rate was estimated at 25% for the control group compared to an anticipated 10% in the experimental group (targeting a 15% absolute reduction). Sample size was calculated using the pooled proportion z-test with a one-sided α of 0.025 and 80% statistical power. Accounting for a 5% overall dropout rate, the required sample size is 214 participants (107 per group).

1.2 Statistical Analysis Population

- **ITT (Intention-to-Treat) Set:**

This includes all participants who were randomized into the treatment groups, regardless of whether they completed the treatment or adhered to the protocol. The analysis is based on the initial group assignment to reflect real-world scenarios.

- **FAS (Full Analysis Set):**

This includes all participants who received the study intervention (questionnaire assessments and radiotherapy). The analysis was conducted according to the initial randomization group but may account for deviations from the protocol.

- **PPS (Per-Protocol Set):**

This includes only those participants who completed the study according to the protocol. The analysis strictly follows the randomized groups and is typically used to assess the treatment effect under ideal conditions.

1.3 Scale Reliability Assessment

Cronbach's α coefficient was used to assess the internal consistency of the patient psychosocial scales (PHQ-9, HADS) and the multi-item dimensions of the QLQ-C30 quality-of-life scale. Single-item measures were unable to calculate Cronbach's α coefficient. All scales were administered at baseline and six follow-up time points: middle of radiotherapy, end of radiotherapy, and 1, 2, 3, and 6 months post-radiotherapy.

© 2025 Pei Y et al. *JAMA Network Open*.

1.4 Methods of Statistical Analysis

Radiotherapy interruption rates, rehospitalizations, and tumor response were analyzed by intention-to-treat, with secondary outcomes (nutrition, psychology, QoL) assessed per protocol principles.

Radiotherapy interruption rates, rehospitalizations, and tumor response were analyzed by intention-to-treat, with secondary outcomes (nutrition, psychology, QoL) assessed per-protocol principles. Continuous variables were presented as mean with standard deviation (SD) for normally distributed data (assessed via Shapiro-Wilk tests) and median with interquartile range (IQR) for non-normally distributed variables, while categorical variables were presented as numbers (%). Continuous baseline variables with normal distribution were compared using independent Student's *t*-tests, while categorical baseline variables were analyzed via χ^2. Radiotherapy interruption rates, rehospitalization rates, and tumor response rates were analyzed by χ^2 tests. The 95% confidence interval (CI) for the rate was calculated using the Wald method. The 95% CI for the mean was calculated using the Z-interval method. The 95% CIs for the duration of radiotherapy interruptions were estimated using the non-parametric bootstrap method. A mixed-effects model was employed to calculate predicted group means, mean differences, 95% CIs, and *P* values in questionnaire scores across follow-up time points and their overall effect. The model incorporated both fixed effects (treatment group, time, and group×time interaction) and random effects (subject), accounting for the correlation structure of repeated measurements. These statistical tests of results were two-sided, with a significance level of $P < 0.05$. Statistical analyses were conducted using R version 4.3.1 (R Foundation for Statistical Computing, Vienna, Austria).

© 2025 Pei Y et al. *JAMA Network Open*.

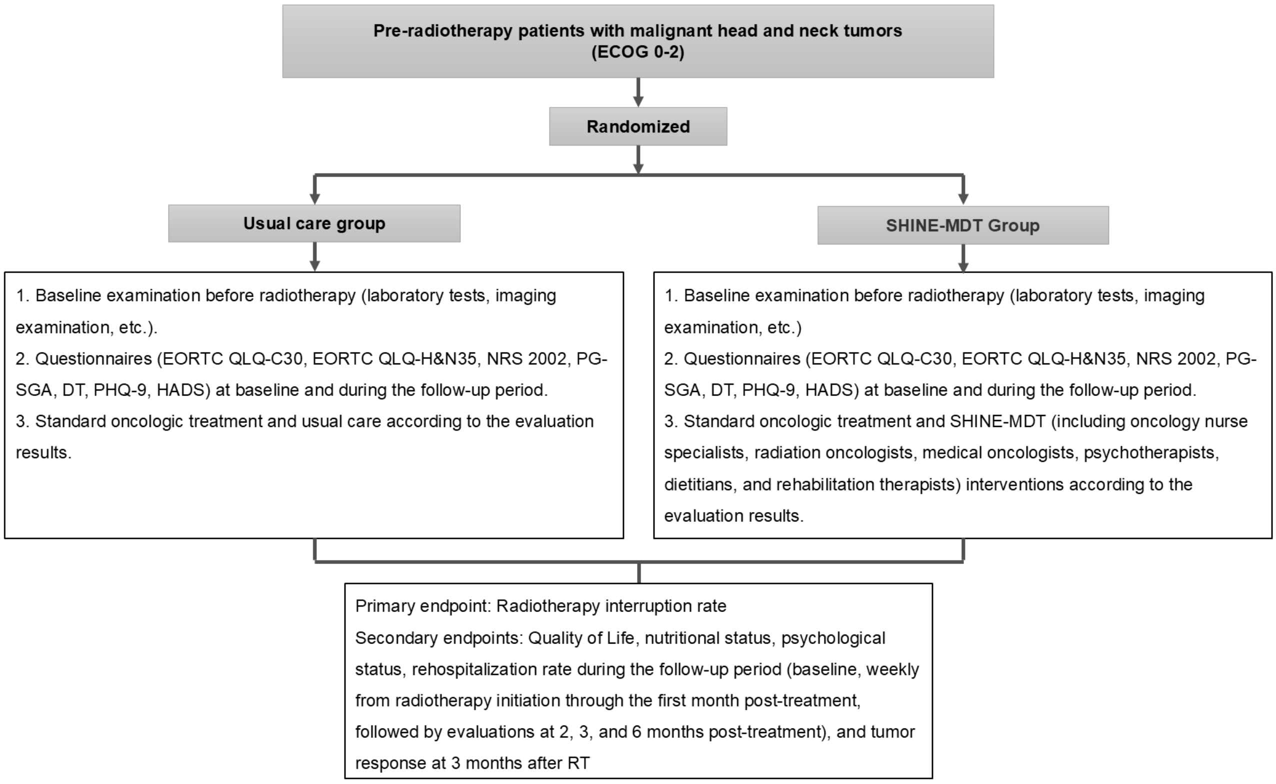

eFigure 1. Overview of the Study Workflow

Abbreviations: ECOG, Eastern Cooperative Oncology Group; SHINE-MDT, Supportive Holistic Interventions by Nurses and Experts via Multidisciplinary Team; EORTC QLQ-C30, European Organization for Research and Treatment of Cancer Quality-of-life Questionnaire Core 30; H&N35, Head and Neck Cancer Module; NRS 2002, Nutrition Risk Screening 2002; PG-SGA, Patient-Generated Subjective Global Assessment; DT, Distress Thermometer; PHQ-9, Patient Health Questionnaire-9; HADS, Hospital Anxiety and Depression Scale; RTI, radiotherapy interruption; RT, radiotherapy.

© 2025 Pei Y et al. *JAMA Network Open*.

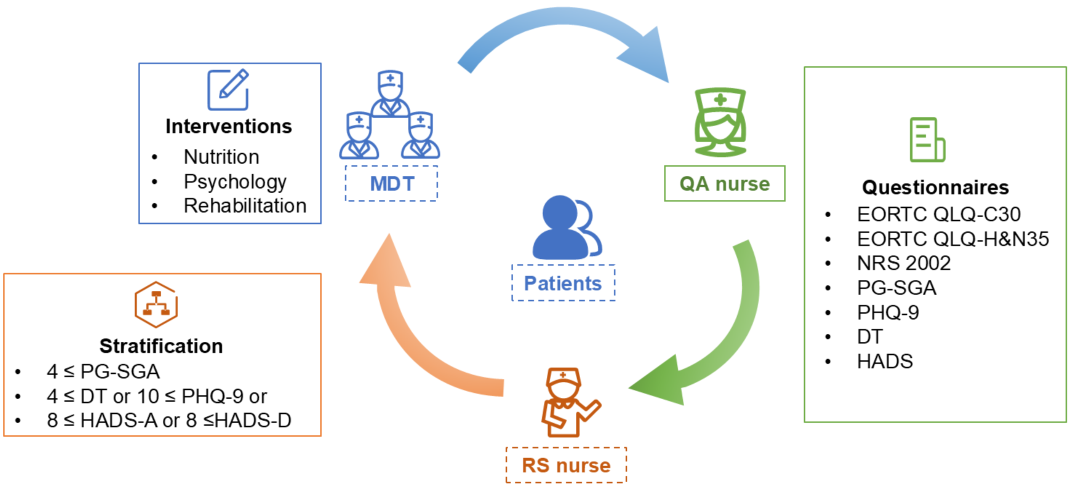

eFigure2. Schematic Representation of the SHINE-MDT Intervention Flow

NOTE. The QA nurse remains blinded to patient group assignments throughout the study and uses standardized questionnaires for evaluations.

Abbreviations: SHINE-MDT, Supportive Holistic Interventions by Nurses and Experts via Multidisciplinary Team; RS nurse, risk-stratified nurse; QA nurse, Questionnaire-assessed nurse; EORTC QLQ-C30, European Organization for Research and Treatment of Cancer Quality-of-life Questionnaire Core 30; H&N35, Head and Neck Cancer Module; NRS 2002, Nutrition Risk Screening 2002; PG-SGA, Patient-Generated Subjective Global Assessment; DT, Distress Thermometer; PHQ-9, Patient Health Questionnaire-9; HADS, Hospital Anxiety, and Depression Scale.

© 2025 Pei Y et al. *JAMA Network Open*.

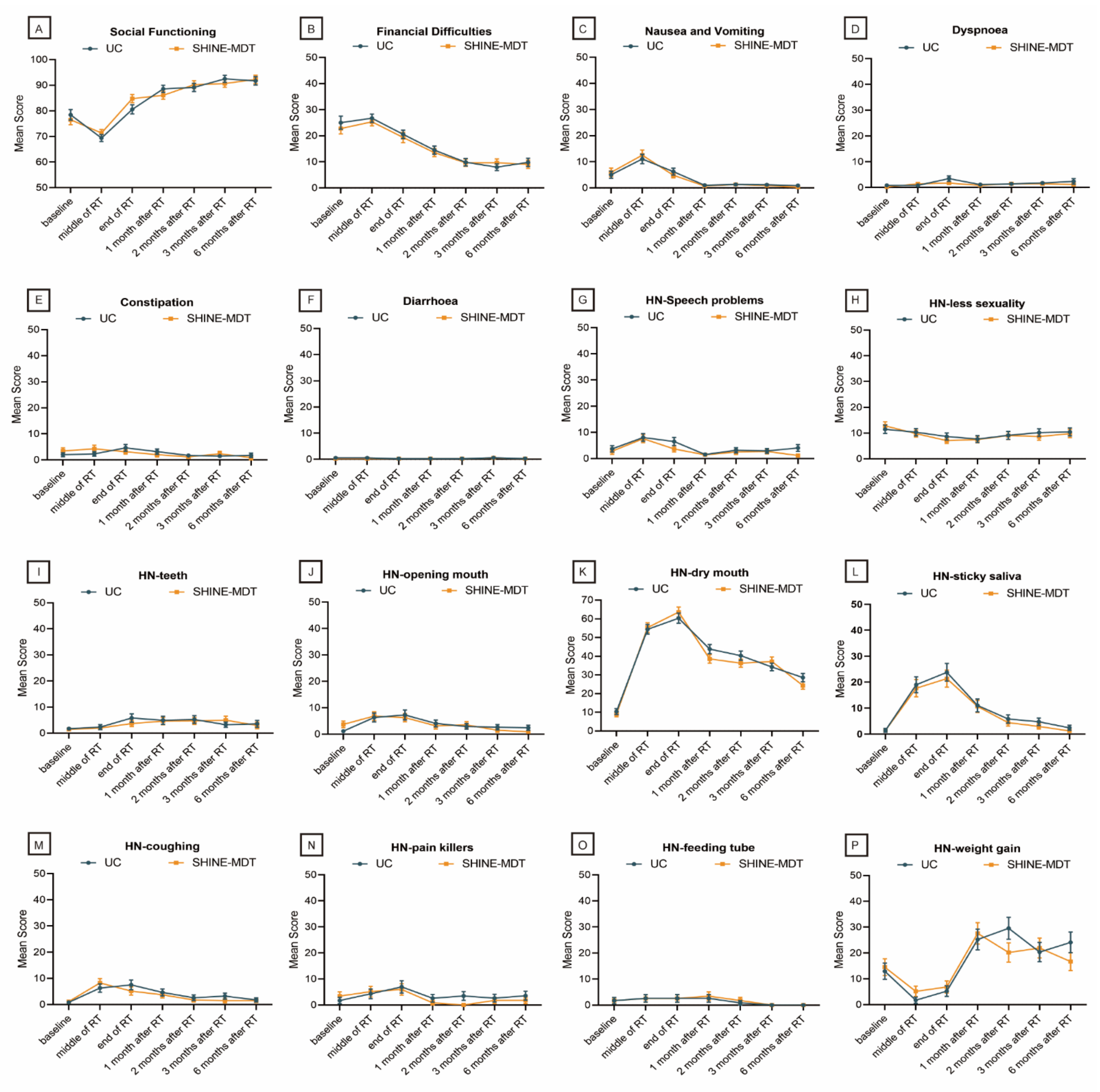

eFigure 3. Quality of Life (EORTC QLQ-C30/QLQ-H&N35) Assessment Scores in UC and SHINE-MDT Groups Over Time.

[A-F] EORTC QLQ-C30; [G-P] EORTC QLQ-H&N35

Abbreviation: UC, usual care, SHINE-MDT, Supportive Holistic Interventions by Nurses and Experts via Multidisciplinary Team.

© 2025 Pei Y et al. *JAMA Network Open*.

eTable 1. Comparison of Radiotherapy Dose Parameters Between Treatment Groups

Parameter	UC Group (n=116)	SHINE-MDT Group (n=117)	Overall (n=233)	P value
**Total RT dose (cGy), Median (IQR)**	6996 (6450-6996)	6996 (6360-6996)	6996 (6420-6996)	0.921
**Maximum point dose (cGy)**				
Brainstem, median (IQR)	4022 (2929-4769)	3907 (2995-4757)	3949 (2961-4763)	0.931
Spinal cord, median (IQR)	3543 (2896-3682)	3462 (3052-3656)	3493 (3011-3673)	0.594
Optic chiasma, median (IQR)	2913 (198-4698)	2399 (258-4524)	2669(245-4617)	0.957
Left optic nerve, median (IQR)	3427 (207-4993)	3022 (236-5063)	3208 (221-5009)	0.936
Right optic nerve, median (IQR)	3571 (210-5002)	3232 (202-5013)	3343 (202-5003)	0.879
Left TMJ, median (IQR)	4772 (1834-6116)	4911 (1879-5975)	4874 (1854-6085)	0.700
Right TMJ, median (IQR)	4440 (1738-5887)	4907 (2669-5774)	4727 (1837-5853)	0.376
**Mean dose (cGy)**				
Oral cavity, median (IQR)	3637 (3059-4262)	3775 (3162-4210)	3690 (3111-4231)	0.814
Larynx, median (IQR)	3578 (2796-3974)	3380 (2688-3804)	3470 (2776-3889)	0.103
Left inner ear, median (IQR)	3402 (713-4243)	2939 (593-4009)	3143 (615-4129)	0.264
Right inner ear, median (IQR)	3194 (707-4136)	3121 (598-3937)	3121 (664-4044)	0.344
Left parotid gland, median (IQR)	1903 (1208-2232)	2012 (1536-2322)	1945 (1346-2300)	0.145
Right parotid gland, median (IQR)	2031 (1471-2281)	2067 (1562-2395)	2047 (1553-2366)	0.438

Abbreviations: IQR = interquartile range; RT = radiotherapy; TMJ = temporomandibular joint

Note: All values presented as median (IQR) unless otherwise specified. *P* values from Mann-Whitney U test comparing two treatment groups.

© 2025 Pei Y et al. *JAMA Network Open*.

eTable 2: Comparison of Consultation Recommendations and Completion Between UC Group and SHINE-MDT Group

Characteristic	UC Group (N =116)	SHINE-MDT Group (N = 117)
**Mean Recommended Consultations (Mean ± SD)**[1]		3.63 ± 1.08
psychology	3.66 ± 1.63	
nutrition	4.87 ± 1.51	
rehabilitation	1.95 ± 0.82	
**Completion ≥1 consultation (n, %)**[2]	29 (25.0%)	117 (100%)
psychology	12 (10.3%)	
nutrition	17 (14.7%)	
rehabilitation	10 (8.6%)	
**Mean Completed Consultations (Mean ± SD)**		
**All patients completed with consultation**[3]		3.34 ± 1.08
psychology	0.14 ± 0.44	
nutrition	0.30 ± 0.76	
rehabilitation	0.09 ± 0.32	
**Patients completed with ≥1 consultation**[4]		3.34 ± 1.08
psychology	1.33 ± 0.49	
nutrition	2.06 ± 0.57	
rehabilitation	1.11 ± 0.30	

Table Notes:

[1]Mean Recommended Consultations: Average number of consultations clinically advised per patient.

[2]Completion ≥1 Consultation: Number (n) and percentage (%) of patients attending ≥1 consultation.

Mean Completed Consultations:

[3]All patients: Average consultations completed (non-attendees counted as 0).

[4]Patients with ≥1 consultation: Average consultations completed among attendees only.

© 2025 Pei Y et al. *JAMA Network Open*.

eTable 3. Reasons for Radiotherapy Interruptions—UC Group vs SHINE-MDT Group

Reasons	UC Group (n = 29)	SHINE-MDT Group (n = 13)
Fatigue	16	2
Leukopenia	8	6
Thrombocytopenia	1	2
Others	3	4

© 2025 Pei Y et al. *JAMA Network Open*.

eTable 4. Longitudinal Changes in Quality of Life, Nutrition Status, and Psychological Status During Radiotherapy in Patients With Malignant Head and Neck Tumor

QLQ-C30（global health status/QoL）

Time Point / Participant numbers (UC vs. SHINE-MDT)	Mean Score (UC vs. SHINE-MDT)	Difference	Standard Error (UC vs. SHINE-MDT)	Median Score（IQR）(UC vs. SHINE-MDT)	p-value
Baseline （116 vs. 117）	76.4 vs. 77.1	-0.7	1.2 vs. 1.3	83.3 (16.7) vs. 83.3 (16.7)	0.685
Middle of RT （116 vs. 117）	66.2 vs. 71.5	-5.3	1.3 vs. 1.3	66.7 (16.7) vs. 66.7 (16.7)	0.002**
End of RT （115 vs. 117）	64.1 vs. 68.6	-4.5	1.3 vs. 1.4	66.7 (16.7) vs. 66.7 (25.0)	0.009**
1 Month After RT （115 vs. 116）	71.7 vs. 75.1	-3.4	1.1 vs. 0.9	66.7 (16.7) vs. 75.0 (16.7)	0.055
2 Months After RT （115 vs. 114）	72.8 vs. 76.5	-3.7	1.1 vs. 0.9	75.0 (16.7) vs. 83.3 (16.7)	0.035*
3 Months After RT （113 vs. 114）	73.1 vs. 77.0	-3.9	1.1 vs. 1.2	75.0 (16.7) vs. 83.3 (16.7)	0.027*
6 Months After RT （112 vs. 114）	73.5 vs. 78.2	-4.7	1.6 vs. 1.3	83.3 (16.7) vs. 83.3 (16.7)	0.008**

QLQ-C30（physical functioning）

Time Point / Participant numbers (UC vs. SHINE-MDT)	Mean Score (UC vs. SHINE-MDT)	Difference	Standard Error (UC vs. SHINE-MDT)	Median Score（IQR）(UC vs. SHINE-MDT)	p-value
Baseline （116 vs. 117）	95.1 vs. 95.9	-0.8	0.8 vs. 0.9	100 (6.7) vs. 100 (6.7)	0.518
Middle of RT （116 vs. 117）	91.0 vs. 91.2	-0.2	1.1 vs. 1.1	93.3 (13.3) vs. 93.3 (13.3)	0.918
End of RT （115 vs. 117）	84.7 vs. 92.5	-7.8	1.0 vs. 0.7	86.7 (10.0) vs. 93.3 (13.3)	<0.001***
1 Month After RT （115 vs. 116）	91.7 vs. 93.6	-1.9	0.8 vs. 0.7	93.3 (13.3) vs. 93.3 (13.3)	0.131
2 Months After RT （115 vs. 114）	91.3 vs. 94.3	-3.0	1.2 vs. 0.8	100 (13.3) vs. 100 (13.3)	0.022*
3 Months After RT （113 vs. 114）	92.2 vs. 95.6	-3.4	0.9 vs. 0.7	93.3 (13.3) vs. 100 (6.7)	0.010*
6 Months After RT （112 vs. 114）	91.7 vs. 95.1	-3.4	1.1 vs. 0.9	93.3 (13.3) vs. 100 (6.7)	0.009**

QLQ-C30（role functioning）

Time Point / Participant numbers (UC vs. SHINE-MDT)	Mean Score (UC vs. SHINE-MDT)	Difference	Standard Error (UC vs. SHINE-MDT)	Median Score（IQR）(UC vs. SHINE-MDT)	p-value
Baseline （116 vs. 117）	94.3 vs. 95.6	-1.3	1.2 vs. 1.1	100 (0) vs. 100 (0)	0.541
Middle of RT （116 vs. 117）	87.6 vs. 88.3	-0.7	2.0 vs. 1.7	100 (16.7) vs. 100 (33.3)	0.756
End of RT （115 vs. 117）	82.8 vs. 89.9	-7.1	1.7 vs. 1.4	83.3 (33.3) vs. 100 (33.3)	0.001**
1 Month After RT （115 vs. 116）	88.1 vs. 92.2	-4.1	1.6 vs. 1.4	100 (25.0) vs. 100 (0)	0.061
2 Months After RT （115 vs. 114）	88.4 vs. 90.8	-2.4	1.7 vs. 1.5	100 (16.7) vs. 100 (16.7)	0.291
3 Months After RT （113 vs. 114）	92.2 vs. 92.1	0.1	1.5 vs. 1.4	100 (0) vs. 100 (16.7)	0.935
6 Months After RT （112 vs. 114）	89.6 vs. 90.9	-1.3	1.8 vs. 1.2	100 (16.7) vs. 100 (16.7)	0.574

QLQ-C30（emotional functioning）

Time Point / Participant numbers (UC vs. SHINE-MDT)	Mean Score (UC vs. SHINE-MDT)	Difference	Standard Error (UC vs. SHINE-MDT)	Median Score（IQR）(UC vs. SHINE-MDT)	p-value
Baseline （116 vs. 117）	93.4 vs. 92.4	1.0	1.1 vs. 1.2	100 (16.7) vs. 100 (16.7)	0.519
Middle of RT （116 vs. 117）	88.6 vs. 91.5	-2.9	1.1 vs. 0.8	91.7 (16.7) vs. 91.7 (16.7)	0.067
End of RT （115 vs. 117）	86.1 vs. 89.9	-3.8	1.4 vs. 1.1	91.7 (25.0) vs. 91.7 (16.7)	0.016*
1 Month After RT （115 vs. 116）	89.9 vs. 94.0	-4.1	1.2 vs. 0.9	100 (16.7) vs. 100 (8.3)	0.009**
2 Months After RT （115 vs. 114）	90.6 vs. 94.1	-3.5	1.1 vs. 1.0	91.7 (16.7) vs. 100 (8.3)	0.029*
3 Months After RT （113 vs. 114）	90.7 vs.93.9	-3.2	1.0 vs. 0.9	91.7 (16.7) vs. 100 (8.3)	0.045*
6 Months After RT （112 vs. 114）	89.9 vs. 93.8	-3.9	1.3 vs. 1.1	91.7 (16.7) vs. 100 (8.3)	0.016*

© 2025 Pei Y et al. *JAMA Network Open*.

QLQ-C30（cognitive functioning）

Time Point / Participant numbers (UC vs. SHINE-MDT)	Mean Score (UC vs. SHINE-MDT)	Difference	Standard Error (UC vs. SHINE-MDT)	Median Score（IQR）(UC vs. SHINE-MDT)	p-value
Baseline （116 vs. 117）	97.6 vs. 99.0	-1.4	0.6 vs. 0.5	100 (0) vs. 100 (0)	0.051
Middle of RT （116 vs. 117）	97.7 vs. 97.0	0.7	0.6 vs. 0.7	100 (0) vs. 100 (0)	0.350
End of RT （115 vs. 117）	96.2 vs. 97.9	-1.7	0.7 vs. 0.5	100 (0) vs. 100 (0)	0.029*
1 Month After RT （115 vs. 116）	99.0 vs. 99.0	0.0	0.4 vs. 0.4	100 (0) vs. 100 (0)	0.990
2 Months After RT （115 vs. 114）	99.0 vs. 98.7	-0.3	0.4 vs. 0.6	100 (0) vs. 100 (0)	0.663
3 Months After RT （113 vs. 114）	99.6 vs. 99.7	-0.1	0.2 vs. 0.2	100 (0) vs. 100 (0)	0.870
6 Months After RT （112 vs. 114）	98.7 vs. 99.1	-0.4	0.6 vs. 0.5	100 (0) vs. 100 (0)	0.570

QLQ-C30（social functioning）

Time Point / Participant numbers (UC vs. SHINE-MDT)	Mean Score (UC vs. SHINE-MDT)	Difference	Standard Error (UC vs. SHINE-MDT)	Median Score（IQR）(UC vs. SHINE-MDT)	p-value
Baseline （116 vs. 117）	78.5 vs. 76.5	-2.0	2.1 vs. 2.0	75.0 (33.3) vs. 66.7 (33.3)	0.394
Middle of RT （116 vs. 117）	69.4 vs. 71.4	2.0	1.4 vs. 1.4	66.7 (0) vs. 66.7 (0)	0.389
End of RT （115 vs. 117）	80.6 vs. 84.8	4.2	1.8 vs. 1.6	66.7 (33.3) vs. 100 (33.3)	0.070
1 Month After RT （115 vs. 116）	88.6 vs. 86.1	2.5	1.4 vs. 1.5	100 (33.3) vs. 100 (33.3)	0.275
2 Months After RT （115 vs. 114）	89.1 vs. 90.2	-1.1	1.6 vs. 1.6	100 (33.3) vs. 100 (33.3)	0.649
3 Months After RT （113 vs. 114）	92.5 vs. 90.6	1.9	1.4 vs. 1.5	100 (0) vs. 100 (16.7)	0.424
6 Months After RT （112 vs. 114）	91.7 vs. 92.3	-0.6	1.7 vs. 1.6	100 (0) vs. 100 (0)	0.813

QLQ-C30（fatigue）

Time Point / Participant numbers (UC vs. SHINE-MDT)	Mean Score (UC vs. SHINE-MDT)	Difference	Standard Error (UC vs. SHINE-MDT)	Median Score（IQR）(UC vs. SHINE-MDT)	p-value
Baseline （116 vs. 117）	5.0 vs. 3.1	1.9	1.1 vs. 1.1	0 (0) vs. 0 (0)	0.337
Middle of RT （116 vs. 117）	16.5 vs. 7.3	9.2	2.0 vs. 1.3	0 (33.3) vs. 0 (11.1)	<0.001***
End of RT （115 vs. 117）	23.3 vs. 13.3	10.0	2.3 vs. 1.7	22.2 (44.4) vs. 0 (22.2)	<0.001***
1 Month After RT （115 vs. 116）	8.2 vs. 5.5	2.7	1.2 vs. 0.7	0 (11.1) vs. 0 (11.1)	0.156
2 Months After RT （115 vs. 114）	8.4 vs. 5.0	3.4	1.4 vs. 1.1	0 (11.1) vs. 0 (0)	0.077
3 Months After RT （113 vs. 114）	6.9 vs. 4.4	2.5	0.9 vs. 0.8	0 (11.1) vs. 0 (0)	0.205
6 Months After RT （112 vs. 114）	7.5 vs. 3.5	4.0	1.5 vs. 1.1	0 (11.1) vs. 0 (0)	0.042*

QLQ-C30（nausea and vomiting）

Time Point / Participant numbers (UC vs. SHINE-MDT)	Mean Score (UC vs. SHINE-MDT)	Difference	Standard Error (UC vs. SHINE-MDT)	Median Score（IQR）(UC vs. SHINE-MDT)	p-value
Baseline （116 vs. 117）	5.0 vs. 6.0	-1.0	1.3 vs. 1.6	0 (0) vs. 0 (0)	0.535
Middle of RT （116 vs. 117）	11.1 vs. 12.5	-1.4	1.8 vs. 2.0	0 (16.7) vs. 0 (16.7)	0.339
End of RT （115 vs. 117）	6.2 vs. 4.8	1.4	1.4 vs. 1.1	0 (0) vs. 0 (0)	0.369
1 Month After RT （115 vs. 116）	1.0 vs. 0.7	0.3	0.5 vs. 0.3	0 (0) vs. 0 (0)	0.860
2 Months After RT （115 vs. 114）	1.3 vs. 1.3	0.0	0.5 vs. 0.7	0 (0) vs. 0 (0)	0.984
3 Months After RT （113 vs. 114）	1.2 vs. 0.9	0.3	0.4 vs. 0.5	0 (0) vs. 0 (0)	0.857
6 Months After RT （112 vs. 114）	0.9 vs. 0.3	0.6	0.5 vs. 0.2	0 (0) vs. 0 (0)	0.713

© 2025 Pei Y et al. *JAMA Network Open*.

QLQ-C30（pain）

Time Point / Participant numbers (UC vs. SHINE-MDT)	Mean Score (UC vs. SHINE-MDT)	Difference	Standard Error (UC vs. SHINE-MDT)	Median Score（IQR）(UC vs. SHINE-MDT)	p-value
Baseline （116 vs. 117）	2.4 vs. 4.0	-1.6	0.8 vs. 1.1	0 (0) vs. 0 (0)	0.455
Middle of RT （116 vs. 117）	19.3 vs. 11.0	8.3	2.3 vs. 1.3	0 (33.3) vs. 0 (16.7)	<0.001***
End of RT （115 vs. 117）	26.2 vs. 17.7	8.5	2.5 vs. 2.1	16.7 (50) vs. 16.7 (33.3)	<0.001***
1 Month After RT （115 vs. 116）	9.6 vs. 5.6	4.0	1.3 vs. 1.0	0 (16.7) vs. 0 (16.7)	0.057
2 Months After RT （115 vs. 114）	7.5 vs. 3.8	3.7	1.3 vs. 0.8	0 (16.7) vs. 0 (0)	0.075
3 Months After RT （113 vs. 114）	5.0 vs. 4.2	0.8	1.2 vs. 1.1	0 (0) vs. 0 (0)	0.723
6 Months After RT （112 vs. 114）	4.5 vs. 2.6	1.9	1.4 vs. 0.9	0 (0) vs. 0 (0)	0.397

QLQ-C30（dyspnoea）

Time Point / Participant numbers (UC vs. SHINE-MDT)	Mean Score (UC vs. SHINE-MDT)	Difference	Standard Error (UC vs. SHINE-MDT)	Median Score（IQR）(UC vs. SHINE-MDT)	p-value
Baseline （116 vs. 117）	0.9 vs. 0.3	0.6	0.6 vs. 0.3	0 (0) vs. 0 (0)	0.550
Middle of RT （116 vs. 117）	0.9 vs. 1.4	0.5	0.6 vs. 0.6	0 (0) vs. 0 (0)	0.561
End of RT （115 vs. 117）	3.5 vs. 1.7	1.8	1.0 vs. 0.7	0 (0) vs. 0 (0)	0.068
1 Month After RT （115 vs. 116）	1.2 vs. 0.9	0.3	0.6 vs. 0.5	0 (0) vs. 0 (0)	0.759
2 Months After RT （115 vs. 114）	1.4 vs. 1.5	-0.1	0.6 vs. 0.6	0 (0) vs. 0 (0)	0.986
3 Months After RT （113 vs. 114）	1.8 vs. 1.5	0.3	0.7 vs. 0.6	0 (0) vs. 0 (0)	0.754
6 Months After RT （112 vs. 114）	2.4 vs. 1.2	1.2	1.1 vs. 0.6	0 (0) vs. 0 (0)	0.218

QLQ-C30（insomnia）

Time Point / Participant numbers (UC vs. SHINE-MDT)	Mean Score (UC vs. SHINE-MDT)	Difference	Standard Error (UC vs. SHINE-MDT)	Median Score（IQR）(UC vs. SHINE-MDT)	p-value
Baseline （116 vs. 117）	15.5 vs. 12.3	3.2	2.3 vs. 2.0	0 (33.3) vs. 0 (33.3)	0.272
Middle of RT （116 vs. 117）	21.9 vs. 13.4	8.5	2.8 vs. 2.3	0 (33.3) vs. 0 (33.3)	0.005**
End of RT （115 vs. 117）	24.6 vs. 15.1	9.5	2.8 vs. 2.0	0 (33.3) vs. 0 (33.3)	0.001**
1 Month After RT （115 vs. 116）	13.9 vs. 8.6	5.3	2.1 vs. 1.6	0 (33.3) vs. 0 (0)	0.077
2 Months After RT （115 vs. 114）	13.9 vs. 8.2	5.7	2.3 vs. 1.7	0 (33.3) vs. 0 (0)	0.055
3 Months After RT （113 vs. 114）	13.0 vs. 7.6	5.4	2.0 vs. 1.5	0 (33.3) vs. 0 (0)	0.072
6 Months After RT （112 vs. 114）	10.1 vs. 5.3	4.8	1.9 vs. 1.6	0 (0) vs. 0 (0)	0.106

QLQ-C30（appetite loss）

Time Point / Participant numbers (UC vs. SHINE-MDT)	Mean Score (UC vs. SHINE-MDT)	Difference	Standard Error (UC vs. SHINE-MDT)	Median Score（IQR）(UC vs. SHINE-MDT)	p-value
Baseline （116 vs. 117）	7.2 vs. 6.8	0.4	1.6 vs. 1.6	0 (0) vs. 0 (0)	0.908
Middle of RT （116 vs. 117）	28.4 vs. 21.7	6.8	3.1 vs. 2.7	33.3 (33.3) vs. 0 (33.3)	0.023*
End of RT （115 vs. 117）	39.1 vs. 29.6	9.5	3.2 vs. 2.9	33.3 (66.7) vs. 0 (66.7)	0.002**
1 Month After RT （115 vs. 116）	9.3 vs. 7.5	1.8	1.8 vs. 1.5	0 (0) vs. 0 (0)	0.553
2 Months After RT （115 vs. 114）	10.1 vs. 6.4	3.7	2.2 vs. 1.6	0 (0) vs. 0 (0)	0.229
3 Months After RT （113 vs. 114）	7.7 vs. 7.9	-0.2	1.5 vs. 1.7	0 (0) vs. 0 (0)	0.933
6 Months After RT （112 vs. 114）	4.8 vs. 2.6	2.2	1.7 vs. 1.0	0 (0) vs. 0 (0)	0.495

QLQ-C30（constipation）

© 2025 Pei Y et al. *JAMA Network Open*.

Time Point / Participant numbers (UC vs. SHINE-MDT)	Mean Score (UC vs. SHINE-MDT)	Difference	Standard Error (UC vs. SHINE-MDT)	Median Score（IQR）(UC vs. SHINE-MDT)	p-value
Baseline （116 vs. 117）	2.0 vs. 3.4	-1.4	0.7 vs. 1.2	0 (0) vs. 0 (0)	0.297
Middle of RT （116 vs. 117）	2.3 vs. 4.3	-2.0	0.9 vs. 1.4	0 (0) vs. 0 (0)	0.144
End of RT （115 vs. 117）	4.6 vs. 3.1	1.5	1.3 vs. 0.9	0 (0) vs. 0 (0)	0.267
1 Month After RT （115 vs. 116）	3.2 vs. 2.0	1.2	1.0 vs. 0.8	0 (0) vs. 0 (0)	0.384
2 Months After RT （115 vs. 114）	1.7 vs. 1.2	0.5	0.7 vs. 0.6	0 (0) vs. 0 (0)	0.676
3 Months After RT （113 vs. 114）	1.5 vs. 2.3	-0.8	0.6 vs. 1.0	0 (0) vs. 0 (0)	0.529
6 Months After RT （112 vs. 114）	1.8 vs. 0.9	0.9	0.9 vs. 0.9	0 (0) vs. 0 (0)	0.510

QLQ-C30（diarrhoea）

Time Point / Participant numbers (UC vs. SHINE-MDT)	Mean Score (UC vs. SHINE-MDT)	Difference	Standard Error (UC vs. SHINE-MDT)	Median Score（IQR）(UC vs. SHINE-MDT)	p-value
Baseline （116 vs. 117）	0.6 vs. 0.3	0.3	0.4 vs. 0.3	0 (0) vs. 0 (0)	0.551
Middle of RT （116 vs. 117）	0.6 vs. 0.3	0.3	0.4 vs. 0.3	0 (0) vs. 0 (0)	0.551
End of RT （115 vs. 117）	0.3 vs. 0.3	0.0	0.3 vs. 0.3	0 (0) vs. 0 (0)	0.996
1 Month After RT （115 vs. 116）	0.3 vs. 0.3	0.0	0.3 vs. 0.3	0 (0) vs. 0 (0)	0.996
2 Months After RT （115 vs. 114）	0.3 vs. 0.3	0.0	0.3 vs. 0.3	0 (0) vs. 0 (0)	0.997
3 Months After RT （113 vs. 114）	0.6 vs. 0.6	0.0	0.4 vs. 0.6	0 (0) vs. 0 (0)	0.993
6 Months After RT （112 vs. 114）	0.3 vs. 0.3	0.0	0.3 vs. 0.3	0 (0) vs. 0 (0)	0.995

QLQ-C30（financial difficulties）

Time Point / Participant numbers (UC vs. SHINE-MDT)	Mean Score (UC vs. SHINE-MDT)	Difference	Standard Error (UC vs. SHINE-MDT)	Median Score（IQR）(UC vs. SHINE-MDT)	p-value
Baseline （116 vs. 117）	25.0 vs. 22.8	2.2	2.5 vs. 2.1	33.3 (33.3) vs. 33.3 (33.3)	0.359
Middle of RT （116 vs. 117）	26.7 vs. 25.4	1.3	1.6 vs. 1.5	33.3 (8.3) vs. 33.3 (33.3)	0.569
End of RT （115 vs. 117）	20.6 vs. 19.4	1.2	1.6 vs. 2.0	33.3 (33.3) vs. 0 (33.3)	0.624
1 Month After RT （115 vs. 116）	14.5 vs. 13.5	1.0	1.5 vs. 1.6	0 (33.3) vs. 0 (33.3)	0.690
2 Months After RT （115 vs. 114）	9.9 vs. 9.6	0.3	1.5 vs. 1.4	0 (33.3) vs. 0 (33.3)	0.946
3 Months After RT （113 vs. 114）	8.0 vs. 9.6	-1.6	1.3 vs. 1.5	0 (0) vs. 0 (33.3)	0.479
6 Months After RT （112 vs. 114）	9.8 vs. 9.1	0.7	1.6 vs. 1.6	0 (33.3) vs. 0 (0)	0.774

QLQ-HN35（pain）

Time Point / Participant numbers (UC vs. SHINE-MDT)	Mean Score (UC vs. SHINE-MDT)	Difference	Standard Error (UC vs. SHINE-MDT)	Median Score（IQR）(UC vs. SHINE-MDT)	p-value
Baseline （116 vs. 117）	2.0 vs. 2.0	0.0	0.4 vs. 0.4	0 (0) vs. 0 (0)	0.991
Middle of RT （116 vs. 117）	21.3 vs. 15.6	5.7	1.5 vs. 1.5	16.7 (18.8) vs. 8.3 (25)	<0.001***
End of RT （115 vs. 117）	25.2 vs. 17.2	8.0	2.0 vs. 1.7	16.7 (37.5) vs. 8.3 (25)	<0.001***
1 Month After RT （115 vs. 116）	11.1 vs. 7.5	3.6	0.9 vs. 0.9	8.3 (16.7) vs. 0 (8.3)	0.022*
2 Months After RT （115 vs. 114）	9.2 vs. 5.6	3.6	1.0 vs. 0.8	8.3 (16.7) vs. 0 (8.3)	0.024*
3 Months After RT （113 vs. 114）	7.0 vs. 5.3	1.7	1.0 vs.0.8	0 (8.3) vs. 0 (8.3)	0.312
6 Months After RT （112 vs. 114）	3.9 vs. 3.1	0.8	0.5 vs. 0.6	0 (8.3) vs. 0 (6.25)	0.645

QLQ-HN35（swallowing）

© 2025 Pei Y et al. *JAMA Network Open*.

Time Point / Participant numbers (UC vs. SHINE-MDT)	Mean Score (UC vs. SHINE-MDT)	Difference	Standard Error (UC vs. SHINE-MDT)	Median Score（IQR）(UC vs. SHINE-MDT)	p-value
Baseline （116 vs. 117）	3.4 vs. 2.8	0.6	0.9 vs. 1.0	0 (0) vs. 0 (0)	0.709
Middle of RT （116 vs. 117）	18.8 vs. 18.2	0.6	1.6 vs. 1.9	16.7 (18.8) vs. 8.3 (33.3)	0.743
End of RT （115 vs. 117）	23.2 vs. 16.5	6.7	2.0 vs. 1.8	16.7 (41.7) vs. 8.3 (25.0)	<0.001***
1 Month After RT （115 vs. 116）	7.5 vs. 7.2	0.3	0.9 vs. 1.0	8.3 (8.3) vs. 0 (8.3)	0.853
2 Months After RT （115 vs. 114）	7.5 vs. 4.9	2.6	1.0 vs. 0.9	8.3 (8.3) vs. 0 (8.3)	0.165
3 Months After RT （113 vs. 114）	4.7 vs. 4.8	-0.1	0.8 vs. 0.9	0 (8.3) vs. 0 (8.3)	0.958
6 Months After RT （112 vs. 114）	4.5 vs. 3.7	0.8	1.3 vs. 0.8	0 (0) vs. 0 (6.25)	0.687

QLQ-HN35（senses problems）

Time Point / Participant numbers (UC vs. SHINE-MDT)	Mean Score (UC vs. SHINE-MDT)	Difference	Standard Error (UC vs. SHINE-MDT)	Median Score（IQR）(UC vs. SHINE-MDT)	p-value
Baseline （116 vs. 117）	4.3 vs. 3.8	0.5	1.0 vs. 0.8	0 (0) vs. 0 (0)	0.863
Middle of RT （116 vs. 117）	38.4 vs. 36.2	2.2	2.5 vs. 2.5	33.3 (37.5) vs. 33.3 (33.3)	0.419
End of RT （115 vs. 117）	56.4 vs. 43.0	13.4	2.6 vs. 2.9	66.7 (50) vs. 33.3 (50.0)	<0.001***
1 Month After RT （115 vs. 116）	34.1 vs. 30.7	3.4	2.1 vs. 1.9	33.3 (33.3) vs. 33.3 (20.8)	0.227
2 Months After RT （115 vs. 114）	25.4 vs. 21.2	4.2	2.1 vs. 1.6	16.7 (33.3) vs. 16.7 (33.3)	0.132
3 Months After RT （113 vs. 114）	17.1 vs. 15.1	2.0	1.4 vs. 1.4	16.7 (33.3) vs. 16.7 (16.7)	0.458
6 Months After RT （112 vs. 114）	8.8 vs. 8.3	0.5	1.2 vs. 1.1	0 (16.7) vs. 0 (16.7)	0.888

QLQ-HN35（speech problems）

Time Point / Participant numbers (UC vs. SHINE-MDT)	Mean Score (UC vs. SHINE-MDT)	Difference	Standard Error (UC vs. SHINE-MDT)	Median Score（IQR）(UC vs. SHINE-MDT)	p-value
Baseline （116 vs. 117）	3.7 vs. 2.8	0.9	1.2 vs. 1.2	0 (0) vs. 0 (0)	0.578
Middle of RT （116 vs. 117）	8.0 vs. 7.6	0.4	1.5 vs. 1.6	0 (11.1) vs. 0 (0)	0.778
End of RT （115 vs. 117）	6.5 vs. 3.7	2.8	1.6 vs. 1.2	0 (0) vs. 0 (0)	0.078
1 Month After RT （115 vs. 116）	1.5 vs. 1.4	0.1	0.7 vs. 0.6	0 (0) vs. 0 (0)	0.946
2 Months After RT （115 vs. 114）	3.2 vs. 2.5	0.7	1.0 vs. 1.0	0 (0) vs. 0 (0)	0.685
3 Months After RT （113 vs. 114）	2.9 vs. 2.7	0.2	0.9 vs. 0.9	0 (0) vs. 0 (0)	0.892
6 Months After RT （112 vs. 114）	4.1 vs. 1.2	2.9	1.3 vs. 0.4	0 (0) vs. 0 (0)	0.074

QLQ-HN35（trouble with social eating）

Time Point / Participant numbers (UC vs. SHINE-MDT)	Mean Score (UC vs. SHINE-MDT)	Difference	Standard Error (UC vs. SHINE-MDT)	Median Score（IQR）(UC vs. SHINE-MDT)	p-value
Baseline （116 vs. 117）	1.4 vs. 2.6	-1.2	0.5 vs. 0.8	0 (0) vs. 0 (0)	0.507
Middle of RT （116 vs. 117）	12.9 vs. 15.0	-2.1	1.5 vs. 1.7	8.3 (25.0) vs. 8.3 (25.0)	0.245
End of RT （115 vs. 117）	20.5 vs. 12.9	7.6	2.2 vs. 1.8	8.3 (41.7) vs. 0 (33.3)	<0.001***
1 Month After RT （115 vs. 116）	9.5 vs. 5.4	4.1	1.1 vs. 1.0	8.3 (16.7) vs. 0 (8.3)	0.027*
2 Months After RT （115 vs. 114）	6.1 vs. 4.3	1.8	1.4 vs. 0.9	0 (0) vs. 0 (0)	0.331
3 Months After RT （113 vs. 114）	1.6 vs. 2.9	-1.3	0.7 vs. 0.9	0 (0) vs. 0 (0)	0.458
6 Months After RT （112 vs. 114）	3.6 vs. 1.3	2.3	1.4 vs. 0.6	0 (0) vs. 0 (0)	0.238

QLQ-HN35（trouble with social contact）

© 2025 Pei Y et al. *JAMA Network Open*.

Time Point / Participant numbers (UC vs. SHINE-MDT)	Mean Score (UC vs. SHINE-MDT)	Difference	Standard Error (UC vs. SHINE-MDT)	Median Score（IQR）(UC vs. SHINE-MDT)	p-value
Baseline （116 vs. 117）	2.5 vs. 2.7	-0.2	0.8 vs. 0.6	0 (0) vs. 0 (0)	0.836
Middle of RT （116 vs. 117）	3.5 vs. 4.5	-1.0	0.8 vs. 1.1	0 (0) vs. 0 (0)	0.318
End of RT （115 vs. 117）	3.5 vs. 1.5	2.0	1.0 vs. 0.6	0 (0) vs. 0 (0)	0.046*
1 Month After RT （115 vs. 116）	0.9 vs. 0.4	0.5	0.4 vs. 0.2	0 (0) vs. 0 (0)	0.641
2 Months After RT （115 vs. 114）	1.8 vs. 1.8	0.0	0.6 vs. 0.7	0 (0) vs. 0 (0)	0.985
3 Months After RT （113 vs. 114）	1.2 vs. 1.3	-0.1	0.5 vs. 0.5	0 (0) vs. 0 (0)	0.867
6 Months After RT （112 vs. 114）	2.7 vs. 0.1	2.6	1.1 vs. 0.1	0 (0) vs. 0 (0)	0.012*

QLQ-HN35（less sexuality）

Time Point / Participant numbers (UC vs. SHINE-MDT)	Mean Score (UC vs. SHINE-MDT)	Difference	Standard Error (UC vs. SHINE-MDT)	Median Score（IQR）(UC vs. SHINE-MDT)	p-value
Baseline （116 vs. 117）	11.4 vs. 12.8	-1.3	1.6 vs. 1.6	0 (33.3) vs. 0 (33.3)	0.519
Middle of RT （116 vs. 117）	10.3 vs. 9.8	0.5	1.4 vs. 1.4	0 (33.3) vs. 0 (16.7)	0.802
End of RT （115 vs. 117）	8.7 vs. 7.1	1.6	1.4 vs. 1.1	0 (16.7) vs. 0 (16.7)	0.447
1 Month After RT （115 vs. 116）	7.7 vs. 7.5	0.2	1.4 vs. 1.3	0 (0) vs. 0 (4.2)	0.921
2 Months After RT （115 vs. 114）	9.1 vs. 9.1	0.0	1.6 vs. 1.5	0 (8.3) vs. 0 (16.7)	0.975
3 Months After RT （113 vs. 114）	10.2 vs. 8.6	1.6	1.5 vs. 1.4	0 (33.3) vs. 0 (16.7)	0.458
6 Months After RT （112 vs. 114）	10.4 vs. 9.8	0.6	1.6 vs. 1.6	0 (16.7) vs. 0 (16.7)	0.769

QLQ-HN35（teeth）

Time Point / Participant numbers (UC vs. SHINE-MDT)	Mean Score (UC vs. SHINE-MDT)	Difference	Standard Error (UC vs. SHINE-MDT)	Median Score（IQR）(UC vs. SHINE-MDT)	p-value
Baseline （116 vs. 117）	1.7 vs. 1.4	0.3	0.7 vs. 0.6	0 (0) vs. 0 (0)	0.864
Middle of RT （116 vs. 117）	2.3 vs. 2.0	0.3	1.0 vs. 0.7	0 (0) vs. 0 (0)	0.862
End of RT （115 vs. 117）	5.8 vs. 3.7	2.1	1.7 vs. 1.2	0 (0) vs. 0 (0)	0.235
1 Month After RT （115 vs. 116）	4.9 vs. 4.6	0.3	1.6 vs. 1.4	0 (0) vs. 0 (0)	0.851
2 Months After RT （115 vs. 114）	5.2 vs. 4.7	0.5	1.5 vs. 1.4	0 (0) vs. 0 (0)	0.762
3 Months After RT （113 vs. 114）	3.2 vs. 5.0	-1.8	1.0 vs. 1.6	0 (0) vs. 0 (0)	0.326
6 Months After RT （112 vs. 114）	3.6 vs. 2.9	0.7	1.3 vs. 1.3	0 (0) vs. 0 (0)	0.724

QLQ-HN35（opening mouth）

Time Point / Participant numbers (UC vs. SHINE-MDT)	Mean Score (UC vs. SHINE-MDT)	Difference	Standard Error (UC vs. SHINE-MDT)	Median Score（QR）(UC vs. SHINE-MDT)	p-value
Baseline （116 vs. 117）	1.1 vs. 3.7	-2.6	0.6 vs. 1.3	0 (0) vs. 0 (0)	0.153
Middle of RT （116 vs. 117）	6.3 vs. 6.8	-0.5	1.7 vs. 1.6	0 (0) vs. 0 (0)	0.773
End of RT （115 vs. 117）	7.2 vs. 6.3	0.9	1.9 vs. 1.6	0 (0) vs. 0 (0)	0.586
1 Month After RT （115 vs. 116）	4.1 vs. 3.1	1.0	1.3 vs. 1.2	0 (0) vs. 0 (0)	0.616
2 Months After RT （115 vs. 114）	2.9 vs. 3.5	-0.6	1.1 vs. 1.3	0 (0) vs. 0 (0)	0.702
3 Months After RT （113 vs. 114）	2.7 vs. 1.5	1.2	1.0 vs. 0.8	0 (0) vs. 0 (0)	0.540
6 Months After RT （112 vs. 114）	2.4 vs. 0.9	1.5	1.0 vs. 0.5	0 (0) vs. 0 (0)	0.436

QLQ-HN35（dry mouth）

© 2025 Pei Y et al. *JAMA Network Open*.

Time Point / Participant numbers (UC vs. SHINE-MDT)	Mean Score (UC vs. SHINE-MDT)	Difference	Standard Error (UC vs. SHINE-MDT)	Median Score（IQR）(UC vs. SHINE-MDT)	p-value
Baseline （116 vs. 117）	10.3 vs. 9.1	1.2	1.6 vs. 1.5	0 (33.3) vs. 0 (0)	0.706
Middle of RT （116 vs. 117）	54.3 vs. 55.3	-1.0	2.6 vs. 2.6	66.7 (33.3) vs. 66.7 (33.3)	0.768
End of RT （115 vs. 117）	60.3 vs. 63.5	-3.2	2.7 vs. 2.7	66.7 (33.3) vs. 66.7 (66.7)	0.322
1 Month After RT （115 vs. 116）	43.8 vs. 38.5	5.3	2.5 vs. 2.3	33.3 (33.3) vs. 33.3 (33.3)	0.107
2 Months After RT （115 vs. 114）	40.3 vs. 36.3	4.0	2.4 vs. 2.2	33.3 (33.3) vs. 33.3 (33.3)	0.217
3 Months After RT （113 vs. 114）	34.2 vs. 37.1	-2.9	2.0 vs. 2.4	33.3 (0) vs. 33.3 (33.3)	0.381
6 Months After RT （112 vs. 114）	28.6 vs. 24.3	4.3	2.2 vs. 2.0	33.3 (33.3) vs. 33.3 (33.3)	0.194

QLQ-C30（sticky saliva）

Time Point / Participant numbers (UC vs. SHINE-MDT)	Mean Score (UC vs. SHINE-MDT)	Difference	Standard Error (UC vs. SHINE-MDT)	Median Score（IQR）(UC vs. SHINE-MDT)	p-value
Baseline （116 vs. 117）	1.4 vs. 1.4	0.0	0.8 vs. 0.6	0 (0) vs. 0 (0)	0.997
Middle of RT （116 vs. 117）	19.0 vs. 17.7	1.3	3.1 vs. 3.3	0 (33.3) vs. 0 (0)	0.669
End of RT （115 vs. 117）	23.8 vs. 21.4	2.4	3.4 vs. 3.2	0 (33.3) vs. 0 (33.3)	0.433
1 Month After RT （115 vs. 116）	11.0 vs. 10.6	0.4	2.5 vs. 2.4	0 (0) vs. 0 (0)	0.899
2 Months After RT （115 vs. 114）	5.8 vs. 4.4	1.4	1.6 vs. 1.3	0 (0) vs. 0 (0)	0.647
3 Months After RT （113 vs. 114）	4.7 vs. 2.9	1.8	1.4 vs. 1.1	0 (0) vs. 0 (0)	0.560
6 Months After RT （112 vs. 114）	2.4 vs. 1.2	1.2	1.0 vs. 0.7	0 (0) vs. 0 (0)	0.699

QLQ-C30（coughing）

Time Point / Participant numbers (UC vs. SHINE-MDT)	Mean Score (UC vs. SHINE-MDT)	Difference	Standard Error (UC vs. SHINE-MDT)	Median Score（IQR）(UC vs. SHINE-MDT)	p-value
Baseline （116 vs. 117）	0.9 vs. 1.1	-0.2	0.6 vs. 0.7	0 (0) vs. 0 (0)	0.871
Middle of RT （116 vs. 117）	6.3 vs. 8.3	-2.0	1.6 vs. 1.7	0 (0) vs. 0 (0)	0.258
End of RT （115 vs. 117）	7.5 vs. 5.1	2.4	1.9 vs. 1.5	0 (0) vs. 0 (0)	0.161
1 Month After RT （115 vs. 116）	4.6 vs. 3.7	0.9	1.4 vs. 1.2	0 (0) vs. 0 (0)	0.600
2 Months After RT （115 vs. 114）	2.6 vs. 1.8	0.8	1.1 vs. 0.8	0 (0) vs. 0 (0)	0.621
3 Months After RT （113 vs. 114）	3.2 vs. 1.5	1.7	1.2 vs. 0.9	0 (0) vs. 0 (0)	0.305
6 Months After RT （112 vs. 114）	1.8 vs. 1.5	0.3	0.8 vs. 0.8	0 (0) vs. 0 (0)	0.853

QLQ-C30（felt ill）

Time Point / Participant numbers (UC vs. SHINE-MDT)	Mean Score (UC vs. SHINE-MDT)	Difference	Standard Error (UC vs. SHINE-MDT)	Median Score（IQR）(UC vs. SHINE-MDT)	p-value
Baseline （116 vs. 117）	4.3 vs. 4.6	-0.3	1.1 vs. 1.3	0 (0) vs. 0 (0)	0.927
Middle of RT （116 vs. 117）	19.8 vs. 16.2	3.6	2.5 vs. 2.4	0 (33.3) vs. 0 (33.3)	0.184
End of RT （115 vs. 117）	21.2 vs. 13.1	8.1	2.7 vs. 2.0	0 (33.3) vs. 0 (33.3)	0.003**
1 Month After RT （115 vs. 116）	18.6 vs. 10.3	8.3	2.3 vs. 1.7	0 (33.3) vs. 0 (33.3)	0.002**
2 Months After RT （115 vs. 114）	14.8 vs. 9.9	4.9	2.0 vs. 1.6	0 (33.3) vs. 0 (33.3)	0.076
3 Months After RT （113 vs. 114）	8.6 vs. 8.1	0.5	1.4 vs. 1.7	0 (0) vs. 0 (0)	0.891
6 Months After RT （112 vs. 114）	6.8 vs. 5.6	1.2	1.8 vs. 1.5	0 (0) vs. 0 (0)	0.654

QLQ-C30（pain killers）

© 2025 Pei Y et al. *JAMA Network Open*.

Time Point / Participant numbers (UC vs. SHINE-MDT)	Mean Score (UC vs. SHINE-MDT)	Difference	Standard Error (UC vs. SHINE-MDT)	Median Score（IQR）(UC vs. SHINE-MDT)	p-value
Baseline （116 vs. 117）	1.7 vs. 3.4	-1.7	1.2 vs. 1.7	0 (0) vs. 0 (0)	0.460
Middle of RT （116 vs. 117）	4.3 vs. 5.1	-0.8	1.9 vs. 2.0	0 (0) vs. 0 (0)	0.721
End of RT （115 vs. 117）	7.0 vs. 6.0	1.0	2.4 vs. 2.2	0 (0) vs. 0 (0)	0.676
1 Month After RT （115 vs. 116）	2.6 vs. 0.9	1.7	1.5 vs. 0.9	0 (0) vs. 0 (0)	0.444
2 Months After RT （115 vs. 114）	3.5 vs. 0.0	3.5	1.7 vs. 0.0	0 (0) vs. 0 (0)	0.125
3 Months After RT （113 vs. 114）	2.7 vs. 1.8	0.9	1.5 vs. 1.2	0 (0) vs. 0 (0)	0.613
6 Months After RT （112 vs. 114）	3.6 vs. 1.8	1.8	1.7 vs. 1.2	0 (0) vs. 0 (0)	0.373

QLQ-C30（nutritional supplements）

Time Point / Participant numbers (UC vs. SHINE-MDT)	Mean Score (UC vs. SHINE-MDT)	Difference	Standard Error (UC vs. SHINE-MDT)	Median Score（IQR）(UC vs. SHINE-MDT)	p-value
Baseline （116 vs. 117）	12.9 vs. 12.8	0.1	3.1 vs. 3.1	0 (0) vs. 0 (0)	0.975
Middle of RT （116 vs. 117）	11.2 vs. 14.5	-3.3	2.9 vs. 3.3	0 (0) vs. 0 (0)	0.339
End of RT （115 vs. 117）	3.5 vs. 10.3	-6.8	1.7 vs. 2.8	0 (0) vs. 0 (0)	0.049*
1 Month After RT （115 vs. 116）	0.9 vs. 7.8	-6.9	0.9 vs. 2.5	0 (0) vs. 0 (0)	0.049*
2 Months After RT （115 vs. 114）	3.5 vs. 8.8	-5.3	1.7 vs. 2.6	0 (0) vs. 0 (0)	0.130
3 Months After RT （113 vs. 114）	2.7 vs. 7.9	-5.2	1.5 vs. 2.5	0 (0) vs. 0 (0)	0.130
6 Months After RT （112 vs. 114）	2.7 vs. 8.8	-6.1	1.5 vs. 2.6	0 (0) vs. 0 (0)	0.077

QLQ-C30（feeding tube）

Time Point / Participant numbers (UC vs. SHINE-MDT)	Mean Score (UC vs. SHINE-MDT)	Difference	Standard Error (UC vs. SHINE-MDT)	Median Score（IQR）(UC vs. SHINE-MDT)	p-value
Baseline （116 vs. 117）	1.7 vs. 1.7	0.0	1.2 vs. 1.2	0 (0) vs. 0 (0)	0.993
Middle of RT （116 vs. 117）	2.6 vs. 2.6	0.0	1.5 vs. 1.5	0 (0) vs. 0 (0)	0.989
End of RT （115 vs. 117）	2.6 vs. 2.6	0.0	1.5 vs. 1.5	0 (0) vs. 0 (0)	0.984
1 Month After RT （115 vs. 116）	2.6 vs. 3.4	-0.8	1.5 vs. 1.7	0 (0) vs. 0 (0)	0.613
2 Months After RT （115 vs. 114）	0.9 vs. 1.8	-0.9	0.9 vs. 1.2	0 (0) vs. 0 (0)	0.609
3 Months After RT （113 vs. 114）	0.0 vs. 0.0	0.0	0.0 vs. 0.0	0 (0) vs. 0 (0)	0.996
6 Months After RT （112 vs. 114）	0.0 vs. 0.0	0.0	0.0 vs. 0.0	0 (0) vs. 0 (0)	0.997

QLQ-C30（weight loss）

Time Point / Participant numbers (UC vs. SHINE-MDT)	Mean Score (UC vs. SHINE-MDT)	Difference	Standard Error (UC vs. SHINE-MDT)	Median Score（IQR）(UC vs. SHINE-MDT)	p-value
Baseline （116 vs. 117）	13.8 vs. 18.8	-5.0	3.2 vs. 3.6	0 (0) vs. 0 (0)	0.289
Middle of RT （116 vs. 117）	41.4 vs. 28.4	13.0	4.6 vs. 4.2	0 (100) vs. 0 (100)	0.006**
End of RT （115 vs. 117）	27.8 vs. 17.1	10.7	4.2 vs. 3.5	0 (100) vs. 0 (0)	0.024*
1 Month After RT （115 vs. 116）	20.9 vs. 11.2	9.7	3.8 vs. 2.9	0 (0) vs. 0 (0)	0.043*
2 Months After RT （115 vs. 114）	17.4 vs. 7.0	10.4	3.5 vs. 2.4	0 (0) vs. 0 (0)	0.032*
3 Months After RT （113 vs. 114）	13.3 vs. 7.0	6.3	3.2 vs. 2.4	0 (0) vs. 0 (0)	0.199
6 Months After RT （112 vs. 114）	5.4 vs. 3.5	1.9	2.1 vs. 1.7	0 (0) vs. 0 (0)	0.726

QLQ-C30（weight gain）

© 2025 Pei Y et al. *JAMA Network Open*.

Time Point / Participant numbers (UC vs. SHINE-MDT)	Mean Score (UC vs. SHINE-MDT)	Difference	Standard Error (UC vs. SHINE-MDT)	Median Score（IQR）(UC vs. SHINE-MDT)	p-value
Baseline （116 vs. 117）	12.9 vs. 14.5	-1.6	3.1 vs. 3.3	0 (0) vs. 0 (0)	0.736
Middle of RT （116 vs. 117）	1.7 vs. 5.1	-3.4	1.2 vs. 2.0	0 (0) vs. 0 (100)	0.474
End of RT （115 vs. 117）	5.2 vs. 6.8	-1.6	2.0 vs. 2.3	0 (0) vs. 0 (0)	0.733
1 Month After RT （115 vs. 116）	25.2 vs. 27.6	-2.4	4.0 vs. 4.1	0 (50) vs. 0 (100)	0.620
2 Months After RT （115 vs. 114）	29.6 vs. 20.2	9.4	4.3 vs. 3.7	0 (100) vs. 0 (0)	0.050
3 Months After RT （113 vs. 114）	20.4 vs. 22.0	-1.6	3.8 vs. 3.8	0 (0) vs. 0 (0)	0.741
6 Months After RT （112 vs. 114）	24.1 vs. 16.7	7.4	4.0 vs. 3.5	0 (0) vs. 0 (0)	0.124

NRS-2002

Time Point / Participant numbers (UC vs. SHINE-MDT)	Mean Score (UC vs. SHINE-MDT)	Difference	Standard Error (UC vs. SHINE-MDT)	Median Score（IQR）(UC vs. SHINE-MDT)	p-value
Baseline （116 vs. 117）	1.4 vs. 1.3	0.03	0.06 vs. 0.05	1 (1) vs. 1 (1)	0.722
Middle of RT （116 vs. 117）	2.3 vs. 2.0	0.3	0.07 vs. 0.07	2 (1) vs. 2 (0)	<0.001***
End of RT （115 vs. 117）	2.8 vs. 2.2	0.6	0.08 vs. 0.06	3 (1) vs. 2 (0)	<0.001***
1 Month After RT （115 vs. 116）	2.6 vs. 2.1	0.5	0.07 vs. 0.03	2 (1) vs. 2 (0)	<0.001***
2 Months After RT （115 vs. 114）	2.3 vs. 2.1	0.3	0.06 vs. 0.03	2 (1) vs. 2 (0)	<0.001***
3 Months After RT （113 vs. 114）	2.2 vs. 2.1	0.1	0.05 vs. 0.03	2 (0) vs. 2 (0)	0.033*
6 Months After RT （112 vs. 114）	1.5 vs. 1.3	0.2	0.07 vs. 0.05	1 (1) vs. 1 (1)	0.007**

PG-SGA

Time Point / Participant numbers (UC vs. SHINE-MDT)	Mean Score (UC vs. SHINE-MDT)	Difference	Standard Error (UC vs. SHINE-MDT)	Median Score（IQR）(UC vs. SHINE-MDT)	p-value
Baseline （116 vs. 117）	4.3 vs. 4.0	0.3	0.3 vs. 0.3	3 (3) vs. 2 (2)	0.591
Middle of RT （116 vs. 117）	10.2 vs. 9.2	1.1	0.3 vs. 0.5	10 (4) vs. 8 (7)	0.024*
End of RT （115 vs. 117）	10.2 vs. 6.9	3.3	0.5 vs. 0.4	10 (8) vs. 6 (6)	<0.001***
1 Month After RT （115 vs. 116）	7.1 vs. 4.4	2.7	0.4 vs. 0.3	6 (7) vs. 3 (2)	<0.001***
2 Months After RT （115 vs. 114）	5.9 vs. 4.3	1.6	0.3 vs. 0.3	5 (5) vs. 3 (3)	<0.001***
3 Months After RT （113 vs. 114）	4.7 vs. 4.0	0.7	0.2 vs. 0.3	4 (2) vs. 3 (2.8)	0.144
6 Months After RT （112 vs. 114）	4.4 vs. 3.2	1.3	0.3 vs. 0.1	4 (3) vs. 3 (1)	0.007**

DT

Time Point / Participant numbers (UC vs. SHINE-MDT)	Mean Score (UC vs. SHINE-MDT)	Difference	Standard Error (UC vs. SHINE-MDT)	Median Score（IQR）(UC vs. SHINE-MDT)	p-value
Baseline （116 vs. 117）	2.3 vs. 2.4	-0.1	0.1 vs. 0.1	2 (2) vs. 2 (3)	0.426
Middle of RT （116 vs. 117）	3.1 vs. 3.0	0.1	0.2 vs. 0.2	3 (3) vs. 3 (3)	0.715
End of RT （115 vs. 117）	4.3 vs. 3.0	1.3	0.1 vs. 0.2	4 (2) vs. 3 (2.3)	<0.001***
1 Month After RT （115 vs. 116）	3.5 vs. 2.5	1.0	0.1 vs. 0.1	3 (1) vs. 2 (3)	<0.001***
2 Months After RT （115 vs. 114）	3.3 vs. 2.5	0.8	0.1 vs. 0.1	3 (2) vs. 2.5 (2)	0.001**
3 Months After RT （113 vs. 114）	3.3 vs. 2.7	0.6	0.1 vs. 0.2	3 (1) vs. 2 (3)	0.002**
6 Months After RT （112 vs. 114）	2.4 vs. 1.9	0.5	0.2 vs. 0.1	2 (2) vs. 1 (2)	0.012*

HADS-A

© 2025 Pei Y et al. *JAMA Network Open*.

Time Point / Participant numbers (UC vs. SHINE-MDT)	Mean Score (UC vs. SHINE-MDT)	Difference	Standard Error (UC vs. SHINE-MDT)	Median Score（IQR） (UC vs. SHINE-MDT)	p-value
Baseline （116 vs. 117）	3.3 vs. 3.1	0.2	0.3 vs. 0.3	3 (5) vs. 3 (4)	0.566
Middle of RT （116 vs. 117）	3.9 vs. 3.6	0.3	0.3 vs. 0.3	3 (5) vs. 3 (5)	0.446
End of RT （115 vs. 117）	7.3 vs. 5.0	2.3	0.4 vs. 0.4	7 (4) vs. 4 (5)	<0.001***
1 Month After RT （115 vs. 116）	5.1 vs. 3.6	1.5	0.3 vs. 0.3	6 (6) vs. 3 (6.5)	<0.001***
2 Months After RT （115 vs. 114）	5.4 vs. 4.0	1.4	0.3 vs. 0.3	6 (3.5) vs. 3 (7)	0.001**
3 Months After RT （113 vs. 114）	5.2 vs. 4.1	1.1	0.3 vs. 0.3	7 (6) vs. 3.5 (6)	0.012*
6 Months After RT （112 vs. 114）	4.0 vs. 3.0	1.0	0.3 vs. 0.3	3 (5) vs. 2 (4)	0.019*

HADS-D

Time Point / Participant numbers (UC vs. SHINE-MDT)	Mean Score (UC vs. SHINE-MDT)	Difference	Standard Error (UC vs. SHINE-MDT)	Median Score（IQR） (UC vs. SHINE-MDT)	p-value
Baseline （116 vs. 117）	4.0 vs. 3.5	0.5	0.3 vs. 0.3	3 (5) vs. 3 (5)	0.329
Middle of RT （116 vs. 117）	4.9 vs. 4.4	0.5	0.4 vs. 0.3	4.5 (5) vs. 4 (5.3)	0.266
End of RT （115 vs. 117）	6.1 vs. 4.5	1.6	0.3 vs. 0.2	6 (4) vs. 4 (3)	<0.001***
1 Month After RT （115 vs. 116）	4.9 vs. 3.5	1.4	0.3 vs. 0.3	5 (5) vs. 3 (5.5)	0.001**
2 Months After RT （115 vs. 114）	4.7 vs. 4.1	0.6	0.3 vs. 0.3	5 (5) vs. 4 (7)	0.095
3 Months After RT （113 vs. 114）	4.4 vs. 4.1	0.3	0.3 vs. 0.3	5 (6) vs. 4 (6)	0.503
6 Months After RT （112 vs. 114）	3.8 vs. 3.0	0.8	0.3 vs. 0.3	3 (5.5) vs. 3 (3)	0.178

PHQ-9

Time Point / Participant numbers (UC vs. SHINE-MDT)	Mean Score (UC vs. SHINE-MDT)	Difference	Standard Error (UC vs. SHINE-MDT)	Median Score（IQR） (UC vs. SHINE-MDT)	p-value
Baseline （116 vs. 117）	1.4 vs. 0.9	0.5	0.2 vs. 0.2	0 (2) vs. 0 (1)	0.098
Middle of RT （116 vs. 117）	2.9 vs. 2.6	0.2	0.3 vs. 0.3	2 (4.3) vs. 1 (5)	0.488
End of RT （115 vs. 117）	3.5 vs. 2.2	1.3	0.3 vs. 0.2	3 (4) vs. 2 (3)	<0.001***
1 Month After RT （115 vs. 116）	1.1 vs. 0.9	0.2	0.2 vs. 0.1	0 (1) vs. 0 (1)	0.633
2 Months After RT （115 vs. 114）	1.2 vs. 0.9	0.3	0.2 vs. 0.2	0 (2) vs. 0 (1)	0.237
3 Months After RT （113 vs. 114）	0.7 vs. 0.6	0.1	0.1 vs. 0.2	0 (1) vs. 0 (0)	0.752
6 Months After RT （112 vs. 114）	1.2 vs. 0.4	0.8	0.3 vs. 0.1	0 (1) vs. 0 (0)	0.012*

© 2025 Pei Y et al. *JAMA Network Open*.

eTable5. Cronbach α Values Across Assessment Time Points

	Baseline	Middle of RT	End of RT	1month after RT	2 month after RT	3 month after RT	6 month after RT
**PHQ-9**	0.753	0.736	0.760	0.720	0.782	0.785	0.789
**HADS-Anxiety**	0.736	0.796	0.859	0.863	0.854	0.868	0.858
**HADS-Depression**	0.738	0.781	0.819	0.810	0.852	0.752	0.848
**QLQ-C30**							
Physical Functioning	0.711	0.832	0.815	0.773	0.824	0.829	0.816
Role Functioning	0.751	0.909	0.893	0.875	0.870	0.899	0.837
Emotional Functioning	0.824	0.757	0.706	0.759	0.775	0.781	0.734
Cognitive Functioning	0.707	0.765	0.736	0.744	0.730	0.784	0.748
Social Functioning	0.904	0.946	0.933	0.948	0.927	0.949	0.912
Global Health Status	0.982	0.957	0.941	0.951	0.965	0.962	0.965
Fatigue	0.890	0.839	0.839	0.866	0.877	0.864	0.844
Nausea/Vomiting	0.875	0.791	0.794	0.709	0.712	0.840	0.787
Pain	0.729	0.790	0.797	0.752	0.716	0.789	0.775

Abbreviations: RT = radiotherapy; PHQ-9 = Patient Health Questionnaire-9; HADS = Hospital Anxiety and Depression Scale; QLQ-C30 = EORTC Quality of Life Questionnaire Core 30

© 2025 Pei Y et al. *JAMA Network Open*.

eTable 6. Comparison of Weight Between UC and SHINE-MDT Groups During and After Radiotherapy

Time Point	UC Group (n=112)	SHINE-MDT Group (n=114)	P-value
Baseline	64.49 ± 12.32	65.32 ± 11.57	0.601
Middle of RT	61.90 ± 11.90	63.59 ± 11.66	0.283
End of RT	60.27 ± 12.31	63.44 ± 11.33	0.045
1 month of RT	61.15 ± 12.07	63.74 ± 11.52	0.101
2 month of RT	61.92 ± 12.32	63.76 ± 11.45	0.245
3 month of RT	62.00 ± 12.40	63.73 ± 11.42	0.277
6 month of RT	63.06 ± 12.27	64.25 ± 11.53	0.452

Note: All weight are presented in kilograms (kg).

P-values were calculated using mixed-effects

© 2025 Pei Y et al. *JAMA Network Open*.

eTable 7. Reasons for Rehospitalization Events—UC Group vs SHINE-MDT Group

Reasons	UC Group (n = 21)	SHINE-MDT Group (n = 9)
Malnutrition	10	2
Infection	3	0
Myelosuppression	3	1
Diabetes	1	3
Others	4	3

© 2025 Pei Y et al. *JAMA Network Open*.

eTable 8. Tumor Response Between UC Group and SHINE-MDT Group

Response	UC Group (n = 74)	SHINE-MDT Group (n = 71)	***P* value**
Complete response	64 (86.5%)	65 (91.5%)	0.227
Partial response	10 (13.5%)	6 (8.5%)	-

Abbreviations: UC, usual care; SHINE-MDT, Supportive Holistic Interventions by Nurses and Experts via a Multidisciplinary Team.

NOTE. Data are n (%). Tumor response assessments were conducted at baseline and 3 months post-radiotherapy in patients with head and neck cancer receiving definitive radiotherapy. Radiographic evaluation included contrast-enhanced CT or MRI of the head and neck, contrast-enhanced CT of the thorax, and abdominal ultrasound. Changes in tumor size were evaluated according to RECIST 1.1 criteria.

© 2025 Pei Y et al. *JAMA Network Open*.
