# Supplementary material for: Multidisciplinary Team Support for Patients With Head and Neck Cancer Receiving Radiotherapy: A Randomized Clinical Trial
Source: JAMA Netw Open. 2025 Dec 15;8(12):e2547590. doi: 10.1001/jamanetworkopen.2025.47590 (PMC12706684; doi:10.1001/jamanetworkopen.2025.47590)
Supplement: Supplement 3. — Data Sharing Statement [file jamanetwopen-e2547590-s003.pdf]

Data Sharing Statement

Pei. Multidisciplinary Team Support for Patients With Head and Neck Cancer Receiving Radiotherapy. *JAMA Netw Open*. Published December 15, 2025.
doi:10.1001/jamanetworkopen.2025.47590

Data
Additional Information: Trial Registry Name: ClinicalTrials.gov Registry URL: https://clinicaltrials.gov Trial Registration Number: NCT05828004
Data available: No

Additional Information
Explanation for why data not available: Data can be requested from the corresponding authors beginning 1 year after publication of the study. De-identified participant data can be available upon approval by the corresponding authors and Sichuan University. A detailed research protocol will be required to evaluate the reasonability of a request for data. The corresponding authors and Sichuan University reserve the right to decide whether or not to share the data based on the materials provided by researchers.
